# Parental vitamin deficiency affects the embryonic gene expression of immune-, lipid transport- and apolipoprotein genes

**DOI:** 10.1038/srep34535

**Published:** 2016-10-12

**Authors:** Kaja H. Skjærven, Lars Martin Jakt, John Arne Dahl, Marit Espe, Håvard Aanes, Kristin Hamre, Jorge M. O. Fernandes

**Affiliations:** 1 National Institute of Nutrition and Seafood Research, NIFES, Norway; 2 Faculty of Biosciences and Aquaculture, Nord University, Norway; 3 Institute of Medical Microbiology, Oslo University Hospital, Norway

## Abstract

World Health Organization is concerned for parental vitamin deficiency and its effect on offspring health. This study examines the effect of a marginally dietary-induced parental one carbon (1-C) micronutrient deficiency on embryonic gene expression using zebrafish. Metabolic profiling revealed a reduced 1-C cycle efficiency in F_0_ generation. Parental deficiency reduced the fecundity and a total of 364 genes were differentially expressed in the F_1_ embryos. The upregulated genes (53%) in the deficient group were enriched in biological processes such as immune response and blood coagulation. Several genes encoding enzymes essential for the 1-C cycle and for lipid transport (especially apolipoproteins) were aberrantly expressed. We show that a parental diet deficient in micronutrients disturbs the expression in descendant embryos of genes associated with overall health, and result in inherited aberrations in the 1-C cycle and lipid metabolism. This emphasises the importance of parental micronutrient status for the health of the offspring.

The nutritional status, and especially micronutrient status, of the parental generation can affect the health of their offspring^1^,^2^,^3^. This effect can result from the general health of both sperm and oocytes, as well as the specific deposition of nutrients and mRNA in the oocytes. After fertilization, the development of the embryo is governed by the interaction of the inherited DNA sequence and its epigenetic status with its environment, including the nutritional supply, complement of mRNA and non-coding RNAs. It has been shown that embryonic development is sensitive to the epigenetic state of the DNA, which can be directly influenced by environmental factors including nutrients, toxins and other stress factors^4^,^5^,^6^. Especially the nutrients of the one carbon (1-C) cycle, which include folate, methionine and choline and the cofactor vitamins B6 and B12^7^, are essential for several vital metabolic processes and regulate a wide spectre of processes like energy metabolism, methylation and transamination reactions. In mammals, perturbation of the 1-C cycle, either through a low 1-C nutrient supply or through depletion/mutation of enzymes has been linked with both developmental deformities and metabolic diseases^4^,^8^,^9^. Nevertheless, no studies have studied a continuous 1-C parental deficiency on embryonic transcriptomes. Transcriptome changes coordinates the complex balance between differentiation and proliferation in the embryos, with an initial phase driven by maternal mRNAs that gradually shifts towards zygotic mRNAs, known as the maternal to zygotic transition^10^. At what developmental stage the major wave of maternal to zygotic transition occurs differs between vertebrates, but as tissues differentiate during gastrulation and later during somitogenesis all vertebrates rely on active zygotic gene transcription. The embryonic period is a sensitive developmental phase for fluctuations in gene expression patterns, and perturbations of embryonic gene expression during this time may result in a permanent and potentially heritable effect^11^.

The importance of 1-C nutrients for growth and embryonic development is well documented^6^, and 1-C supplementation reduces the prevalence of mammalian embryo deformities like neural tube defects and cardiovascular abnormalities^12^,^13^. Folate circulates in the body mainly as tetrahydrofolate (THF) and donates a methyl group to re-methylate homocysteine to methionine^14^. In this process, vitamin B12 is a cofactor. However, deficiency in 1-C nutrients leads to accumulation of cytotoxic homocysteine. Homocysteine can be detoxified through the trans-sulfuration pathway, which requires vitamin B6, and leads to increased levels of cystathionine and eventually glutathione^4^. World Health Organization (WHO) reports low blood concentrations of 1-C nutrients occurring across population groups, both in developing and industrialized countries, and recommends national surveys to measure blood vitamin concentrations, as well as diets including supplements or fortified foods to ensure that physiological requirements are met^15^. The consequences of deficiency are widespread and span from anemia to reduced cognitive function and developmental defects. WHO are actively seeking more research on 1-C deficiency across generations and on its effect on gene expression^15^.

Here we test the hypothesis that parental nutritional status can affect gene regulation during embryonic development. We have previously shown that abiotic environmental factors can affect zygotic gene expression after the mid-blastula transition and especially during somitogenesis^16^. Our previous experience from zebrafish feeding trials^17^ and data from the nutritional aquaculture society^18^,^19^,^20^ allowed us to design a defined feed with a balanced nutritional profile including all the essential macronutrients, micronutrients and minerals for zebrafish. Furthermore, we modified this feed to contain slightly below the requirement levels of 1-C nutrients, and to allow for the study of the effect of parental 1-C deficiency on F_1_ embryonic gene expression through RNA sequencing in this potent vertebrate model organism. We found that a continuous 1-C parental deficiency reduced the fecundity and massively affects the embryonic transcriptomes related to immune response, blood coagulation, redox regulation. In addition, we give clear evidence for changes in transcripts that regulate lipid transportation, like apolipoproteins, which also regulate developmental processes.

## Materials and Methods

### Experimental design and diets

**Figure 1:**
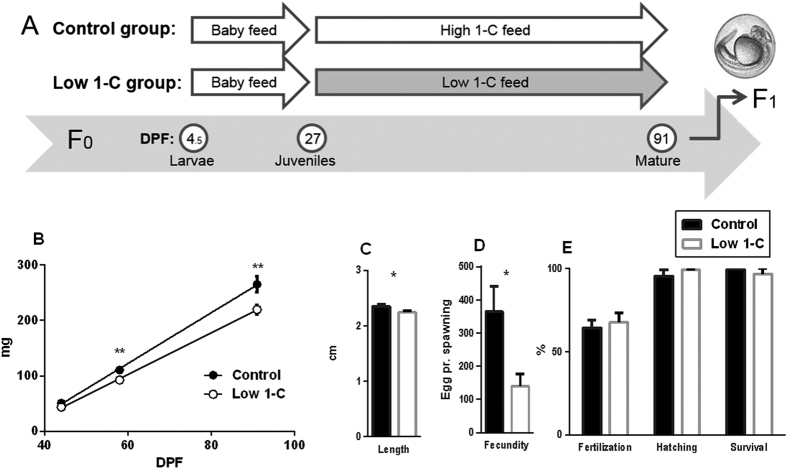
(**A**) Experimental design: F_0_ generation zebrafish were divided into Control group or low 1-C group fed either a high 1-C feed or a low 1-C feed from 27 DPF until mature, and F_1_ generation collected for transcriptome analysis. (**B,C**) F_0_ body mass from 27 to 91 DPF (**B)** and length at 91 DPF **(C)** for F_0_ generation fed either a control diet or a low 1-C diet. The data are represented as means ± SEM from 10 independent tanks (4 randomly selected fish from each tank) from each feed group. (**D)** F_0_ fecundity, measured as number of eggs spawned for each crossing. Data originates from nine independent crossings per feed. (**E**) % fertilization, hatching and survival until 5 DPF of F_1_ embryos in control and low 1-C embryos. Significant differences (see Material and methods section) between feed groups are marked by asterisks (*p < 0.05, **p < 0.01).

Experimental design is given in Fig. 1A. The experimental diets were designed, either slightly below the requirement level (low 1-C diet) or above (control diet) and in accordance with the requirement levels given for carp^19^. Diet formulations and chemical analysis of the feeds are listed in Table 1. The ingredients for the protein blend were ground (using Retch GM200), sieved (Retch AS200, ensuring a particle size ≤80 μm), and mixed for 20 minutes (Electrolux mix master). The protein blend ingredients were a gift from BIOMAR AS, Norway. Dextrin, cellulose, lecithin, mineral mix, vitamin mix and sucrose were mixed (dry ingredients). The 1-C nutrients; folate, vitamin B12 (cyanocobalamin), vitamin B6, methionine, and choline were added to the control feed, whereas the low 1-C feed contained only the amount present in the protein blend raw materials (Table 1a). All vitamins were obtained from Vilomix Norway AS, Norway. All combined dry ingredients were carefully mixed with the oil blend (Table 1a) containing tocopherol (obtained from BAFS Brattvåg, Norway) and MilliQ water (65 °C) containing agar (Sigma) and astaxanthin (G. O. Johnsen AS, Norway) to make a homogenous paste. The two feed pastes, control and low 1-C, were dried for 72 hours at 42 °C, strained through a 1,5 × 1 mm mesh and sieved to obtain the desired feed pellet sizes (<200 μm, 200–400 μm, 400–510 μm, 510–750 μm, 750–1000 μm), and stored at −30 °C until feeding.

### Chemical analyses

Vitamin B12 and folate were analysed microbiologically both in feeds and in 44 days post fertilization (DPF) fish using *Lactobacillus delruceckii spp. lactis* and *Lactobacillus rhamnosus*, respectively, as previously described^21^. The quantity of vitamin B6 in the feeds (pyridoxine, pyridoxal and pyridoxamine) were measured by UPLC as described^22^. Free amino acids were analysed in the feeds on an ultra-performance liquid chromatography system (Waters Acquity UPLC BEH C18 column) as described earlier^18^. Choline was measured fluorometrically in the feeds using a choline quantification kit according to the manufacturer’s procedure (BioVision, USA). The feeds 1-C nutrient and free amino acid composition are given Table 1b.

### Ethical considerations

The zebrafish feeding trial comply with the terms and guidelines of the Norwegian Regulation on Animal Experimentation and European Community Directive 86/609/EEC. The Norwegian Food Safety Authority approved the experimental protocol for zebrafish feeding trials performed in NIFES’s laboratory (division No. 54, reference 2012/145126).

### Feeding trial and fish husbandry

We standardized the operating procedures for mating, handling and feeding wildtype AB zebrafish (Table S1). The two diet groups were randomly assigned to ten replicate tanks at 15 DPF containing 60 larvae. The experimental feeds were given twice a day from 27 DPF until sampling (feed pellet size: 27 DPF; <200 μm, 30 DPF; 200–400 μm, 44 DPF; 400–510 μm, 65 DPF; 510–750 μm, 90 DPF; 750–1000 μm) with an initial individual wet weigh per fish of 9.4 mg (±4.8). For 27–44 DPF the fish were fed the experimental diets *ad libitum*, thereafter they were fed a restrictive diet of 7% and 5% of the tank total biomass from 44 and 90 DPF, respectively. The fish were kept 12/12 h light/dark day cycles and water temperature 27 ± 1 °C.

### Sampling and crossing

At 44, 58, and 91 DPF 4 fish from each of the ten tanks per feed, were anesthetised in MS222 (0.5 min in 50 mg/100 mL), briefly dried on tissue paper and weighed (standard length measures only at 91 DPF) prior to either sampling or returned to the tanks. At 44 DPF, 40 fish from each tank were flash frozen in liquid N2 (metabolite sampling of whole fish was performed between the 2^nd^ and 3^rd^ hour of light in the 12 h/12 h light/dark day cycle), homogenized using a pre-cooled mortar (−78 °C) and stored in −80 °C prior to further analysis for folate, vitamin B12 and metabolomics.

At 80 DPF, three pairs of parents originating from independent feeding tanks from each diet were crossed in separate mating tanks to obtain F_1_ embryos. The F_1_ embryos were handled as described for F_0_ embryos. The fecundity, fertilization, hatching and survival rates were monitored for one mating pair per tank, as described^23^. For gene expression studies of the F_1_ we collected 30 pooled embryos at 1 DPF (26 somite stage, staged accordingly^24^) from each mating pair (embryo gene expression sampling was performed between the 3^rd^ and 4^th^ hour of light in the 12 h/12 h light/dark day cycle). The samples were flash frozen and stored at −80 °C for further analysis.

### Metabolic profiling

Homogenized zebrafish (44 DPF) from six tanks for each dietary treatment were extracted and prepared for global metabolic profiling analysis using Metabolon, Inc. (Durham, NC, USA) standardised procedures. Briefly, whole fish homogenates were subjected to methanol extraction; extracts were split into four aliquots and processed for analysis by ultrahigh performance liquid chromatography/mass spectrometry (UHPLC/MS) in the positive, negative and polar ion modes. Metabolites were identified by automated comparison of ion features to a reference library of chemical standards followed by visual inspection for quality control. To determine statistical significance, Welsh’s two-factor t-test was performed on protein-normalized data in ArrayStudio (Omicsoft) to compare data between experimental groups; p < 0.05 was considered significant. In addition, an estimate of the false discovery rate (q-value)^25^, with q < 0.1 used as an indication of high confidence of a result. Main pathway and sub pathway enrichment scores were calculated relative to the full set of 566 detected metabolites. MetaboLync Cytoscape Plugin was used to generate pathway classification network of regulated metabolites.

### RNA extraction, mRNA sequencing and RTqPCR verification of F_1_ embryos

Exactly 30 embryos were defrosted in 1 mL Trizol reagent and homogenized using a Precellys 24 homogenizer at 3 × 15 s at 6000 rpm with 10 sec intervals. RNA extractions were performed according to the Trizol manufacturer’s protocol (Invitrogen, USA). The quantity and quality of RNA were assessed using a Nanodrop ND-1000 UV Spectrophotometer (NanoDrop Technologies, USA) and an Agilent 2100 Bioanalyser (Agilent Technologies, USA), respectively.

mRNA-sequencing (mRNA-seq) was performed with Illumina Hi-seq run (Rapid run, 50 cycles, single-read, 5 nM concentration) using the dUTP protocol to give strand specificity. Reads were mapped against the zebrafish genome (GRCz10, Ensembl 82) using STAR^26^ with the following options: –outFilterMultimapNmax 5, –outFilterMismatchNoverLmax 0.05, and –outFilterIntronMotifs RemoveNoncanonicalUnannotated. On average, each sample had 35 million reads, of which an average 25 million mapped uniquely. The mapping statistics for each sample are given in Supplementary Figure S1. Expression levels and differential expression were estimated using Cufflinks and Cuffdiff, respectively (v.2.2.1), and both were run using options for multi-read correction and with gene annotation from Ensembl (GRCz10, Ensembl 82). We also included a GTF file to mask ribosomal genes and pseudogene counts. The resulting data was analysed and visualised using Perl scripts and R.

Analysis of enrichment of functional groups was performed using the DAVID 6.7^27^. We used human orthologues as input to DAVID, and the full set of identified orthologues was used as background. Genes belonging to specific Gene Ontology (GO) annotations were identified using the org.Hs.eg.db Bioconductor package (Carlson M. *org.Hs.eg.db: Genome wide annotation for Human*. R package version 3.2.3^28^) and mapped back to zebrafish orthologues. Apolipoprotein genes were identified by searching for gene identifiers containing the apo string.

To verify the RNA sequencing results we performed reverse transcription followed by quantitative real-time PCR (RT-qPCR) as described^29^. Gene expression within individual samples were normalized using *ef1a* and *tuba1* as reference genes. GeNorm^30^ was used to calculate the mean normalized gene expression of three target genes: methionine adenosyltransferase 1, alpha (*mat1a*), catalase (*cat*) and apolipoprotein A-II (*apoA2*). Full names, gene abbreviations, accession numbers, forward and reverse sequences, primer amplicon sizes and qPCR efficiencies are listed in Table S2.

### Statistical treatment

Statistical calculations comparing the two feed groups were performed in Statistica 12 (Statsoft, Inc., USA) for weight measures, while GraphPad Prism 6 (GraphPad Software, USA) was used for analyses of length, fecundity, fertilization, hatching, survival, folate and vitamin B12. Differences in weight were assessed by repeated measure ANOVA followed by Tukey’s *post hoc* test. Levene’s test was applied to test for homogeneity in variance between the groups. Unpaired t-tests were performed for length data, fecundity, fertilization, hatching, survival, folate and vitamin B12. For statistical testing of metabolomics and RNA sequencing results, see corresponding sections above. For all tests, differences were accepted as significant at p < 0.05.

## Results

### Low 1-C feed affected the growth and fecundity of F_0_

Decreasing the level of 1-C nutrients in the feed had a significant effect on F_0_ growth both in terms of weight and length (Fig. 1B,C). Wet weight was significantly lower in the low 1-C feed group at both 58 DPF (p < 0.006) and 91 DPF (p < 0.008). Similarly, at 91 DPF the fish fed low 1-C feed were on average approximately 5% shorter than the control group (P < 0.03). Moreover, reduction in 1-C nutrients had a drastic effect on F_0_ egg production with mean egg number reduced by more than half (365 ± 75 to 139 ± 38 eggs per crossing, p = 0.011, Fig. 1D). However, egg quality did not seem to be affected, as fertilization, hatching and survival rates were not significantly different between feeding groups (Fig. 1E).

### The low 1-C diet affected the body composition of 1-C nutrients in F_0_

**Figure 2:**
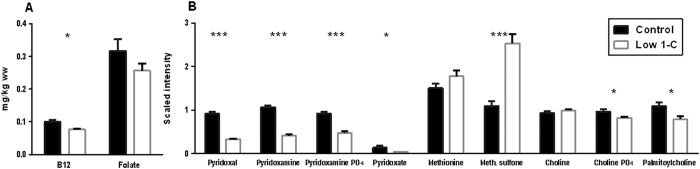
F_0_ 1-C nutrient composition measured in zebrafish (44 DPF) fed either a control or low 1-C diet from 27 DPF until sampling. (**A**) Vitamin B12 and folate concentrations (mg/kg wet weight). (**B**) Scaled intensity measured by metabolic profiling of vitamin B6 (pyridoxal, pyridoxamine, pyridoxamine PO_4_, pyridoxate), methionine, methionine sulfone, choline, choline PO_4_, palmitoylcholine. Values represents pooled samples of 20 fish from 3 (vitamin B12 and folate) or 6 (metabolic profiling) independent tanks from each diet. Significant differences (see Material and methods section) between feed groups are marked by asterisks (*p < 0.05, **p < 0.01, ***p < 0.001).

To verify specifically if the level of 1-C nutrient in the feed had an effect on the overall 1-C composition in F_0_ fish at 44 DPF, we used chemical composition analyses of vitamin B12 and folate (n = 3, Fig. 2A) as well as metabolic profiling (n = 6, Fig. 2B) to assay levels of vitamin B6 (pyridoxal, pyridoxamine, pyridoxamine phosphate, pyridoxate), methionine (methionine and methionine sulfone) and choline (choline, choline PO4, palmitotylcholine). The low 1-C diet had a measurable effect on some, but not all, of the 1-C nutrients. Consistent with a lower dietary intake, levels of vitamin B12 and B6 were detected at significantly lower levels in the low 1-C group (Fig. 2A,B). We did not detect a difference in the level of folate and methionine, though the oxidized form, methionine sulfone, was present at approximately twice the levels of the controls in the low 1-C group (p < 0.001). A decreased supplementation of choline did not affect its level in the two different groups, but both choline phosphate and palmitoylcholine were reduced significantly in the low 1-C group (p < 0.03, both).

### Low 1-C diet affected the amino acid regulation in F_0_

**Figure 3:**
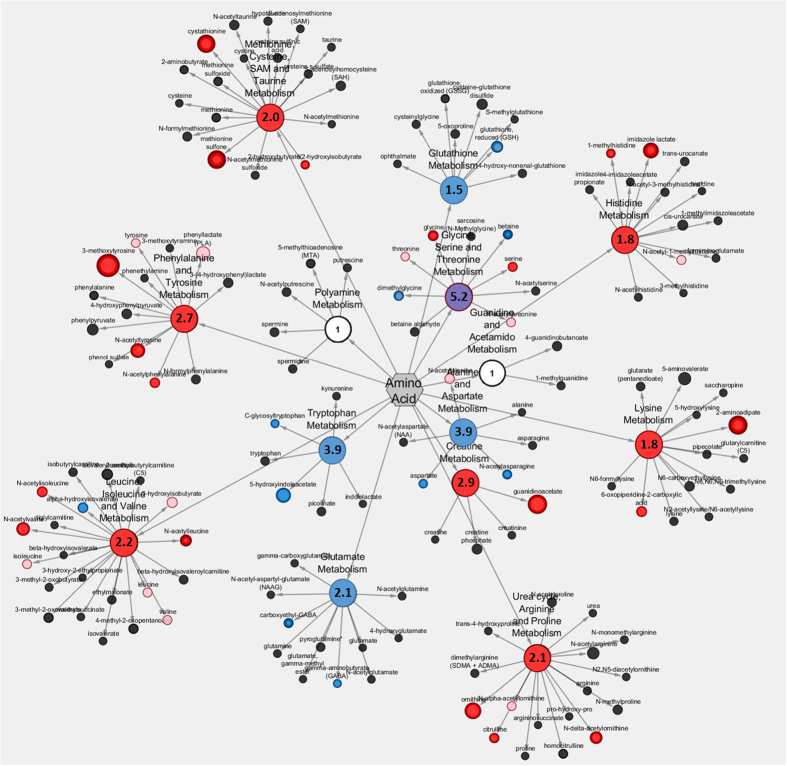
Amino acid sub pathway enrichment score map from metabolic profiling. Red colour indicate higher levels, whereas blue colour indicate lower levels of significant different amino acids related metabolites in low 1-C feed group compared to control. Dot size indicate differences in intensity fold change. For calculations of sub pathway enrichment scores see Table S3.

Metabolic profiling detected a total of 566 metabolites but only the levels of 20 and 27 metabolites decreased and increased, respectively in the low 1-C group (Fig. S2). To identify the main and sub pathways enriched in the affected metabolites we calculated enrichment scores for pathways with significant changes (Table S3). The major effect of the low 1-C feed were the metabolites associated with free amino acid regulation. We observed changes in metabolites tightly connected to the metabolic flow of the 1-C cycle, including glycine, serine and threonine metabolism, methionine, cysteine, SAM and taurine metabolism and glutathione metabolism (Fig. 3). In addition we also observed an effect on nucleotide and lipid regulation (Table S3). The low 1-C feed did not alter the level of oxidized glutathione, S-adenosylhomocysteine or S-adenosylmethionine (Fig. S2A).

Increased levels of several acetylated amino acids are consistent with the overall low levels of 1-C nutrients. The N-acetyl moiety of the three branched chain amino acids, leucine, isoleucine, valine (Fig. S2B), were increased in the low 1-C fish. These acetylated amino acids are catabolized via beta-alanine, which was also elevated (Fig. S2C). In addition, the 1-C deficient fish had higher levels of both citrulline and ornithine (intermediate metabolites of the urea cycle) and lipid endocannabinoid compounds (linoleoyl-, oleoyl- and steraoyl etahanolamide) (Fig. S2C), and a lower level of the inhibitory neurotransmitter gamma-aminobutyrate (GABA) and carboxyethyl-GABA (Fig. S2C).

### F_0_ diet has a striking effect on F_1_ mRNA expression during early development

**Figure 4:**
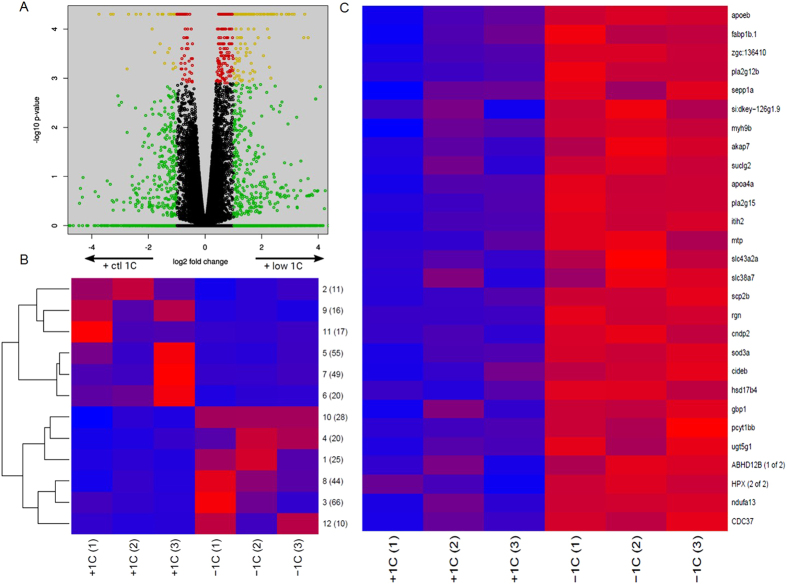
(**A**) Gene expression in low 1-C embryos compared to control embryos. Differential expression as reported by Cufflinks. x-axis: log2 ratios (control / low 1-C) of expression values, y-axis: raw p-values. Green indicates a fold change larger than 2, red a q-value of less than 0.05, yellow both of these and black neither of these. (**B**) Mean expression levels of clusters of differentially expressed genes. Genes identified as differentially expressed by Cuffdiff were divided into 12 clusters by k-means clustering of their levels across the replicate series. Each row of the heat map displays the cluster mean expression scaled by row. The cluster number (1–12) and number of differentially expressed genes are indicated at each row. (**C**) The most consistent k-means cluster of mRNA expression level (cluster 10, 28 genes) from control (+1C) and low 1-C (−1C) F_1_ embryos. Expression levels are indicated by colour codes, with blue to red indicating min to max expression level for each gene, respectively. Each row of the heat map with its given gene name ID displays gene expression level of differentially expressed genes.

We identified a total of 364 genes as differentially expressed (FDR < = 0.05, Fig. 4A and Table S4) in the F_1_ embryos as a result of parental 1-C nutrient status. Of these, 172 transcripts were expressed at lower levels, whereas 192 transcripts had a higher expression level in the low 1-C embryo group (Fig. 4A). We mapped both up- and down-regulated genes to human orthologues and used DAVID (see methods) to assess the enrichment for molecular and physiological functions. Of the up- and down-regulated genes, 145 and 116 had orthologues, respectively. The up-regulated genes (Table 2 (complete list of significant GO terms: Table S5)) were strongly enriched for biological process GO terms associated with inflammation (Fig. S3A), blood coagulation, processing of both proteins and lipids as well as redox regulation. These genes were also enriched for molecular function GO terms associated with peptidases and their regulation, redox activity and lipid transport, largely mirroring the enrichments observed for biological processes GO terms. More than a third of the up-regulated genes were associated with the extracellular region cellular component GO terms, in addition, we observed a strong enrichment for vesicle lumen and platelet alpha granules GO terms (Table 2). We observed a similar enrichment for the corresponding KEGG pathways; complement and coagulation cascade as well as for the PPAR signalling pathway (Table S6).

We used k-means clustering to divide all DEGs into 12 clusters according to their expression pattern across the 6 samples (Fig. 4B). We performed gene enrichment analyses for all individual clusters, but these did not reveal additional or stronger associations between gene expression and function than that observed for the full set of genes.

**Figure 5:**
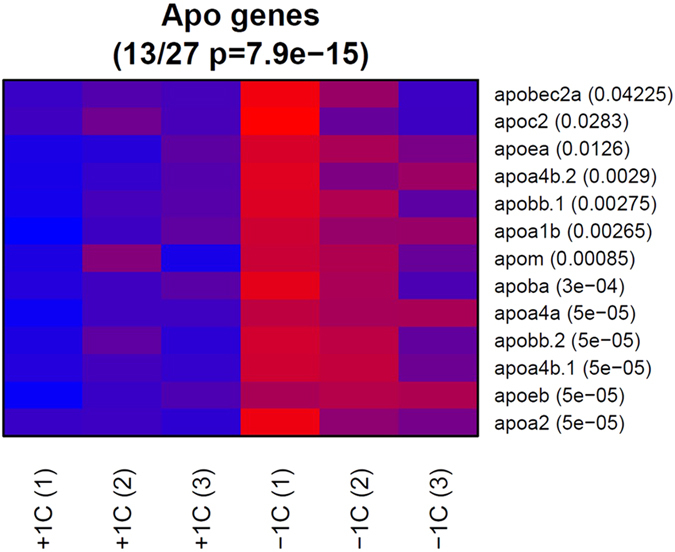
mRNA expression levels of significantly affected apolipoproteins sequenced from control (+1C) and low 1-C (−1C) F_1_ embryos. The low 1-C F_1_ embryos have significantly higher expression levels than control embryos. Expression levels are indicated by colour codes, with blue to red indicating min to max expression level for each gene, respectively. P-values are indicated next to the gene names.

Of the 12 DEG clusters, one cluster (cluster 10, containing 28 genes) showed a strongly homogeneous up-regulation in all replicate low 1-C samples (Fig 4C). This cluster was enriched for the same set of biological processes found for the whole set of up-regulated genes, but interestingly, 9 of these genes were associated with the processing of lipids. As such, we looked in more detail at the gene expression of 114 lipid transporters and 27 apolipoproteins. 26 of the lipid transporter (Fig. S3B) and 13 of the apolipoprotein (Fig. 5) genes were called as differentially expressed with a p-value of less than 0.05; in both cases all of these were up-regulated in the low 1-C samples (p = 1e-18 and p = 8e-15, respectively by hypergeometric test for over-representation) suggesting an overall shift in lipid processing and transport. In addition, we identified several genes in the 1-C metabolism that were upregulated in the low 1-C embryos (Fig. S3C). Except from the redox regulation (Fig. S4), we did not observe any significant enrichment for genes supressed in the low 1-C group. To verify the RNA sequencing results we did RT-qPCR of three selected genes (*mat1a*, *cat*, *apoA2*). All genes revealed the same expression pattern as seen with the RNA sequencing results (Fig. S5).

## Discussion

We have investigated the effects of a dietary 1-C deficiency throughout larval growth and adult stages, both on the F_0_ recipients of the diet and their F_1_ progeny. We observed a reduction in the overall growth of the F_0_ generation, and a major effect in fecundity with a more than two-fold reduction in egg numbers. However, fertilisation and development rates of the offspring were not affected by the diet. Metabolic profiling of the F_0_ generation revealed a reduced nutritional capacity of the 1-C cycle, as we observed changes in both 1-C nutrients and in metabolites tightly connected to the metabolic flow of the 1-C metabolism^4^,^14^,^31^,^32^,^33^. Although we did not observe an effect on the gross morphology of the embryos, we were able to see a clear effect of the parental diet in the developing embryos at the transcriptome level. Genes whose expression was upregulated were enriched for immune- and blood coagulation/haemostasis functions as well as redox regulation, protein and lipid metabolism.

Metabolic profiling in the F_0_ generation showed that the 1-C deficiency affects the tissue levels of a number of downstream metabolites directly involved in the 1-C cycle, which might explain the lower growth of this F_0_ generation compared to their counterparts fed sufficient 1-C nutrients. In addition, the 1-C deficiency influences a range of previously described biological processes such as cellular proliferation, processing of amino acids and proteins, methylation potential, redox regulation and neurotransmitter synthesis^4^,^14^,^31^,^32^,^33^. Increased levels of several acetylated amino acids are consistent with the overall low levels of 1-C nutrients and suggest a deactivation of transamination reactions and protein synthesis, which specifically utilise vitamin B6 and B12 as cofactors^34^. This leads to an amino acid conversion into their corresponding ketoacid, N-acetyl derivative, which is catabolized via beta-alanine, which was elevated in the low 1-C group. High levels of methylhistidine, imidazole lactate, and beta-alanine indicate a low capacity for protein deposition, and are symptomatic of muscle protein degradation to provide energy^18^. Both betaine, and its product dimethylglycine, were decreased in the low 1-C group, either as an effect of increased breakdown, or due to its function as a methyl donor, or as a direct effect of lower choline levels in the feed. Low level of betaine indicate a limitation of choline^4^, and betaine serves as an important methyl donor and methylate homocysteine to methionine, thus producing dimethylglycine. Furthermore, increased levels of glycine and serine in the low 1-C group have previously been linked with a deficiency in the folate cycle as well as a lower capacity for de novo purine synthesis as both amino acids control the methyl group delivery to tetrahydrofolate^14^.

Glutathione is the most important redox regulator and is present in high concentrations in all cells^35^, and the 1-C metabolism is directly linked to the redox regulation through the trans-sulfuration pathway^4^. The lower level of glutathione and increased level of cystathionine indicate a reduced capacity to ameliorate oxidative stress, accomplished by the accumulation of the oxidized form of methionine, the methionine sulfone. Cystathionine has previously been used as a biomarker for 1-C nutrient deficiency^36^. Again this indicates a reduced metabolic capacity in the 1-C cycle.

It has been demonstrated that dietary modifications in fish can lead to inflammation and associated hyper-proliferation causing an increase in tumour formation^37^. The most obvious effect on embryonic gene expression observed was the altered complement and coagulation cascades KEGG pathway and increase in the transcript levels of genes associated with biological process GO term of inflammatory response, redox regulation and blood coagulation/haemostasis, suggesting that the parental dietary modification may affect immune system activity in offspring. This raises the possibility that a parental metabolic imbalance can in itself give rise to an inflammatory response or affect the development of both the vascular and immune systems. At the 26 somite stage the embryo has an open vascular systemic circulation with the first blood vessel differentiation and blood island assembly^32^,^38^,^39^, while the first immune cells exists as myeloid precursor cells produced by the inner cell mass^40^). Both systems, are at this stage, actively differentiating and remodelling and may be sensitive to the parental nutritional status.

Embryogenesis is a time of tissue growth and a high cellular turnover rate where redox regulation affects the balance between proliferation and differentiation rate^41^, where a mild oxidative stress can stimulate differentiation, rather than proliferation^42^,^43^. In the F_1_ embryos we observed an enrichment in GO term related to oxidation reduction. The DEGs associated with redox regulation include several genes that directly control cellular redox regulation in the cells, such as thioredoxin, catalase, superoxide dismutase, and in addition are linked to free amino acid regulation like cysteine dioxygenase, proline dehydrogenase, and phenylalanine hydrolase. Thioredoxin for instance is especially important during embryo development, as knockdown studies are embryonic lethal^44^, and in addition it is important for cardiovascular health^45^. Our metabolic profiling also suggested an increased oxidative stress in the parental low 1-C group, and this result is in line with previously reported studies that have shown that altered maternal micronutrients result in increased oxidative stress in the offspring^46^ and the DEGs in this study correspond to altered gene expression patterns in oxidative stressed murine placenta, namely *noxo1a*, *mat1a*, and *apoe*^47^.

Single nucleotide polymorphisms of genes encoding important enzymes of the 1-C cycle have been associated with severe offspring consequences and influence susceptibility to vascular disease, neural tube defect, colon cancer and acute leukemia^4^. One of them, the *mthfr* was expressed at lower level in low 1-C embryos and this gene encodes an important enzyme that catalyses the folate cycle^48^. Several other 1-C related genes revealed higher expression levels in low 1-C embryos. Earlier stage- and tissue specific expression data and knockdown studies have indicated that 1-C related genes have specific roles during liver, myotome, kidneys or brain development^14^,^48^,^49^,^50^, but are also known from the mammalian literature to be important for protein turnover, DNA synthesis, DNA methylation potential or redox regulation^4^.

Our data suggest that a parental low 1-C diet alters the lipid transportation in F_1_ embryos, and especially apolipoproteins, as 13 (including ApoB, ApoE, ApoA-1, ApoA-IV) out of 27 apolipoproteins genes sequenced were significantly upregulated in the low 1-C group. The apolipoprotein mRNAs encode the protein component of lipoproteins that enables packing and trafficking of lipids in both lymphatic and circulatory systems, while other groups of apolipoproteins serve as cofactors for enzymes and receptor ligands. Expression patterns of apolipoproteins are highly regulated during embryonic development^51^, vital for embryonic lipid transportation in both zebrafish and humans^51^,^52^,^53^. Specifically, in zebrafish, apoB synthesis is vital for correct angiogenesis^53^, while apoA-4a expression is sensitive to feed intake^51^. Interestingly, a maternally disturbed 1-C metabolism in mice also noted a change in expression of one apolipoprotein, the ApoAI, in the offspring^54^, which together with this study indicate a clear relationship between parental micronutrient status and changes in lipid transportation in the offspring.

There has been a natural decline in dietary 1-C nutrients, leading to recommended feed fortification programs (e.g. folate supplements of flour)^15^ for human nutrition and increased inclusion of 1-C nutrients in plant based aquaculture diets^19^. Even though this study shows that a combined 1-C deficiency in the parental generation resulted in less growth and a lower fecundity, it did not lead to embryo deformities as has been shown in mammalian systems^55^,^56^. This might be explained by the specific 1-C nutrient inclusion in the feed. According to previous observations^18^,^19^,^20^, we designed our feed (using feed raw materials with naturally low levels of 1-C nutrients) to be marginally below or at the requirement levels for all the 1-C nutrients studied. This was done to avoid a complete deficiency in any single 1-C nutrient which might be compensated by an increased turnover in alternative metabolic mechanisms, but instead to result in an overall reduced rate of the 1-C metabolism. However, this implies that we cannot exclude the possibility that our observations are primarily caused by one or a subset of the reduced nutrients.

Our observations support the hypothesis that a deficiency in 1-C micronutrients^57^ or an artificially disrupted 1-C cycle (methotrexate treated embryos)^48^ challenges embryonic development. In mammals, exposure to parental dietary stress, like malnutrition or toxins, at critical developmental periods are known to influence the next generation with consequences beyond early development^58^,^59^. Our recent studies in zebrafish have revealed that the parental diet affects the liver transcriptome and the DNA methylation landscape in the next generation (F_1_) of adult males, with concomitant changes in phenotype (Skjærven, unpublished). In conclusion, it is clear that a deficit in 1-C nutrients in the parental diet can affect not only the expression of genes involved in 1-C metabolism in F_1_ embryos but also the expression of numerous genes that regulate embryonic development and that are involved in immune response and blood coagulation. In addition, we give clear evidence that a low 1-C parental diet alter lipid transport through gene expression changes that regulate developmental processes, such as angiogenesis.

## Additional Information

**How to cite this article**: Skjærven, K. H. *et al*. Parental vitamin deficiency affects the embryonic gene expression of immune-, lipid transport- and apolipoprotein genes. *Sci. Rep.* **6**, 34535; doi: 10.1038/srep34535 (2016).

## Supplementary Material

Supplementary Information

Supplementary Table S1

Supplementary Table S2

## Figures and Tables

**Table 1:** Diet formulation and chemical analysis.

Feed	Control	Low-1C
**a) Diet formulation (g/kg)**		
Protein blend 1	768	768
Oil blend 2	80	80
Agar	1	1
Dextrin	46,44	50,00
Cellulose	19,25	19,25
Lecitin	20	20
Mineral mix 3	50	50
Vitamin mix 4	10	10
Astaxanthin	0,003	0,003
Sucrose	1	1
Tocopherol	0,75	0,75
Choline (50%) 5	1	0
Vitamin B12 (0.1%)	1	0
Folate	0,011	0
Vitamin B6	0,020	0
Methionine	2,533	0
**b) Diet chemical analysis of 1-C nutrients and amino acids (g/kg)**		
Choline	1.903	1.254
Vitamin B12 (mg/kg)	0,649	0,009
Folate (mg/kg)	12,51	0,32
Vitamin B6 (mg/kg)	23,21	1,86
Methionine	9,41	5,79
Serine	23,25	22,93
Arginine	29,52	30,00
Glycine	17,25	17,36
Aspartate	48,54	47,45
Glutamine	94,57	93,70
Threonine	16,41	16,41
Alanine	20,45	20,10
Proline	26,61	26,67
Lysine	28,37	27,24
Tyrosine	15,20	15,95
Valine	21,09	21,32
Isoleucine	19,67	19,94
Leucine	38,20	38,32
Phenylalanine	24,35	25,44

1 Protein blend: Fishmeal 5%, Krillmeal 1%, Soya protein concentrate 6,2%, Corn 5%, Wheat 7.5%, Wheat gluten 13%, Pea protein 49.9%, Field peas 12,5%. 2 Oil blend: Fish oil 1%, Rapeseed oil 60%, Flaxseed oil 25%, Arachidonic acid oil 5%. 3 Mineral mix: CaHPO4 x 2H2O 55.25%, CoCl2 x 6H2O 0.01%, CuSO4 x 5H2O 0.04%, K2SO4 27.62%, KI 0.09%, MgSO4 x 7H2O 9.21%, MnSO4 x H2O 0.09%, NaCl 5.29%, Se-yeast 0.37%, ZnSO4 x 7H2O 0.92%, FeSO4 x 7H2O 1.10%. 4 Vitamin mix: VitA 0.2%, VitD3 0.04%, VitE (50% stock (S)) 2%, VitK (50% S) 0.1%, VitC (35% S) 3.5%, Ascorbic acid 10%, Thiamin 0.15%, Riboflavin (80% S), 0.19%, Niacin 2,00%, Inositol 4%, CA-pantothenat 0.6%, Biotin (2% S) 0.5%. Choline 10,00% in control, 0% in low 1-C. Protein blend (carrier) 66.72% in control. 76.72% in low 1-C. 5 Choline: Added as 10% of the vitamin mix of the control feed.

**Table 2:** Gene ontology analyses for biological processes, molecular function and cellular components for differentially expressed genes in low 1-C F_1_ embryos compared to control F_1_ embryos.

Biological process	GoTerms*	Genes*	P-value range**	Benjamini range**
Inflammatory and immune response	31	68	3.5E-12–4.3E-4	5.0E-9–9.5E-3
Blood coagulation, haemostasis, wound healing	6	27	1.6E-7–3.0E-4	2.6E-5–7.4E-3
Processing of proteins, carboxylic- and amino acids	16	58	1.9E-7–3.0E-4	2.5E-5–7.4E-3
Lipid transport and metabolic processing	19	37	4.1E-7–3.8E-4	3.7E-5–8.8E-3
Oxidation reduction	1	20	2.5E-4	1.6E-4
**Molecular function**	**GoTerms**	**Genes**	**P-value range**	**Benjamini range**
Peptidase inhibitor activity	4	13	1.3E-9–1.7E-5	3.3E-12–1.7E-7
Oxidoreductase activity	3	21	3.6E-5–6.6E-3	4.7E-4–2.4E-4
Lipid transport	2	8	4.1E-5–7.8E-3	6.4E-7–3.1E-4
Other significant processes	8	39	9.3E-4–9.3E-3	1.7E-5–3.8E-4
**Cellular component**	**GoTerms**	**Genes**	**P-value range**	**Benjamini range**
Extracellular space and region	3	47	8.9E-18–2.4E-10	1.9E-15–1.8E-8
Vesicle lumen, platelet–and secretory granules	4	8	8.7E-8–5.9E-6	4.6E-6–1.8E-4

In each ontology, the most enriched groups of significant GO terms, with the number of GO terms, genes and p-value range and Benjamini range are listed. *For a complete list of significantly differentially expressed genes enriched in each GO term please see Table S5.

**The p-value and Benjamini ranges refers to the highest and lowest GO term specific value for each group of GO terms.
