# Supplementary material for: Parental vitamin deficiency affects the embryonic gene expression of immune-, lipid transport- and apolipoprotein genes
Source: Sci Rep. 2016 Oct 12;6:34535. doi: 10.1038/srep34535 (PMC5059634; doi:10.1038/srep34535)
Supplement: Supplementary Information [file srep34535-s1.pdf]

**Supplementary material for:**

# Parental vitamin deficiency affects the embryonic gene expression of immune-, lipid transport- and apolipoprotein genes

**Kaja H. Skjærven[1*], Lars Martin Jakt[2], John Arne Dahl[3], Marit Espe[1], Håvard Aanes[3], Kristin Hamre[1] and Jorge M.O. Fernandes[2]**

[1]*National Institute of Nutrition and Seafood Research, NIFES, Norway*

[2]*Faculty of Biosciences and Aquaculture, Nord University, Norway*

[3]*Institute of Medical Microbiology, Oslo University Hospital, Norway*

[*]Corresponding author:

Dr. Kaja Helvik Skjærven

National Institute of Nutrition and Seafood Research (NIFES),

PO Box 2029 Nordnes,

5817 Bergen, Norway

Tel.:+4741458362; fax: +4755905299.

E-mail address: ksk@nifes.no

**Supplementary figures:**

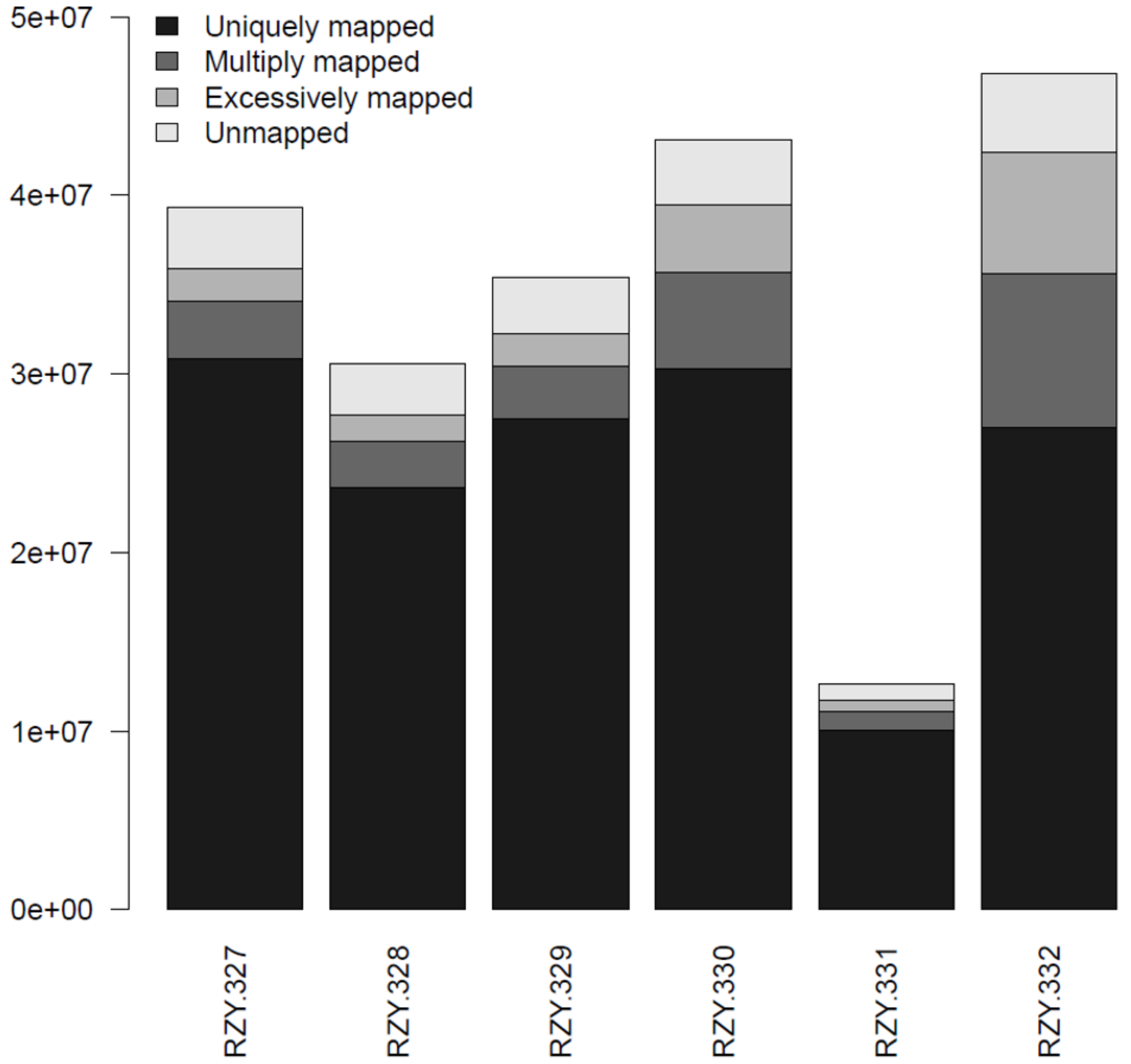


**Figure S1: Mapping statistics from STAR for Illumina Hi-sequencing** run indicating total reads, uniquely mapped and excessively mapped reads for each of the three independent samples from each feeding group. Low 1-C: RZY.327-329, Control: RZY.330-332.

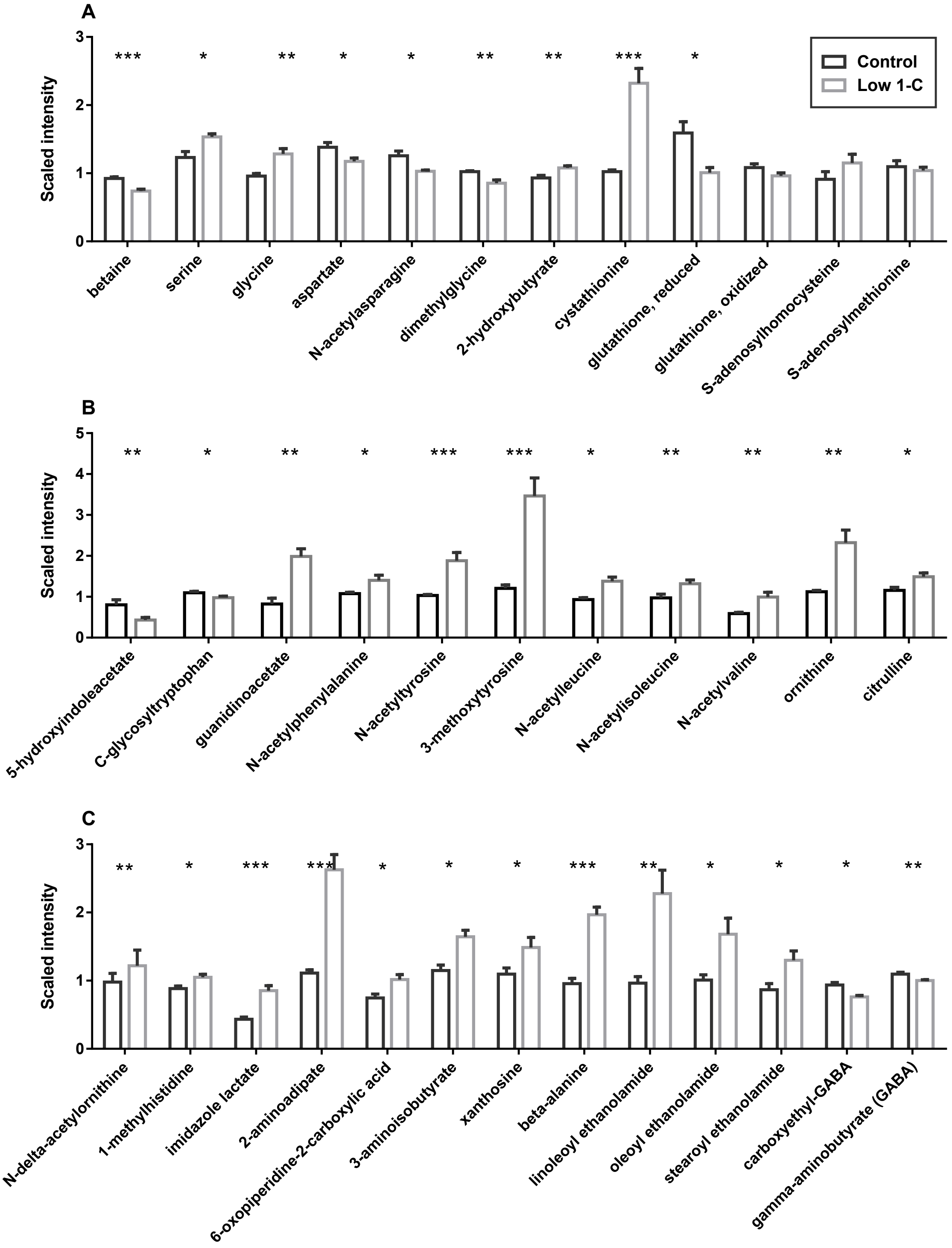


**Figure S2: $F_0$ metabolic profiling of significantly altered metabolites zebrafish (44 DPF) fed control or low 1-C diet. A:** Scaled intensity of metabolites involved in the 1-C pathway. Oxidized glutathione, S-adenosylhomocysteine, and S-adenosylmethionine were not significantly altered. **B:** Scaled intensity of significantly altered metabolites related to amino acid metabolism and **C:** related to amino acid (continuation from B), endocannabinoid (linoleoyl-, oleoyl- and steraoyl etahanolamide) and neurotransmitter (gamma-aminobutyrate (GABA) and carboxyethyl-GABA) metabolism and regulation. Values represent pooled samples of 20 fish from 6 independent tanks from each diet. Significant differences between feed groups are marked by asterisks (* = $p < 0.05$, ** = $p < 0.01$, *** = $p < 0.001$). For pathway enrichment, fold changes, and q-values see Table S3, for pathway classification network interactions of amino acids, see Fig. 3.

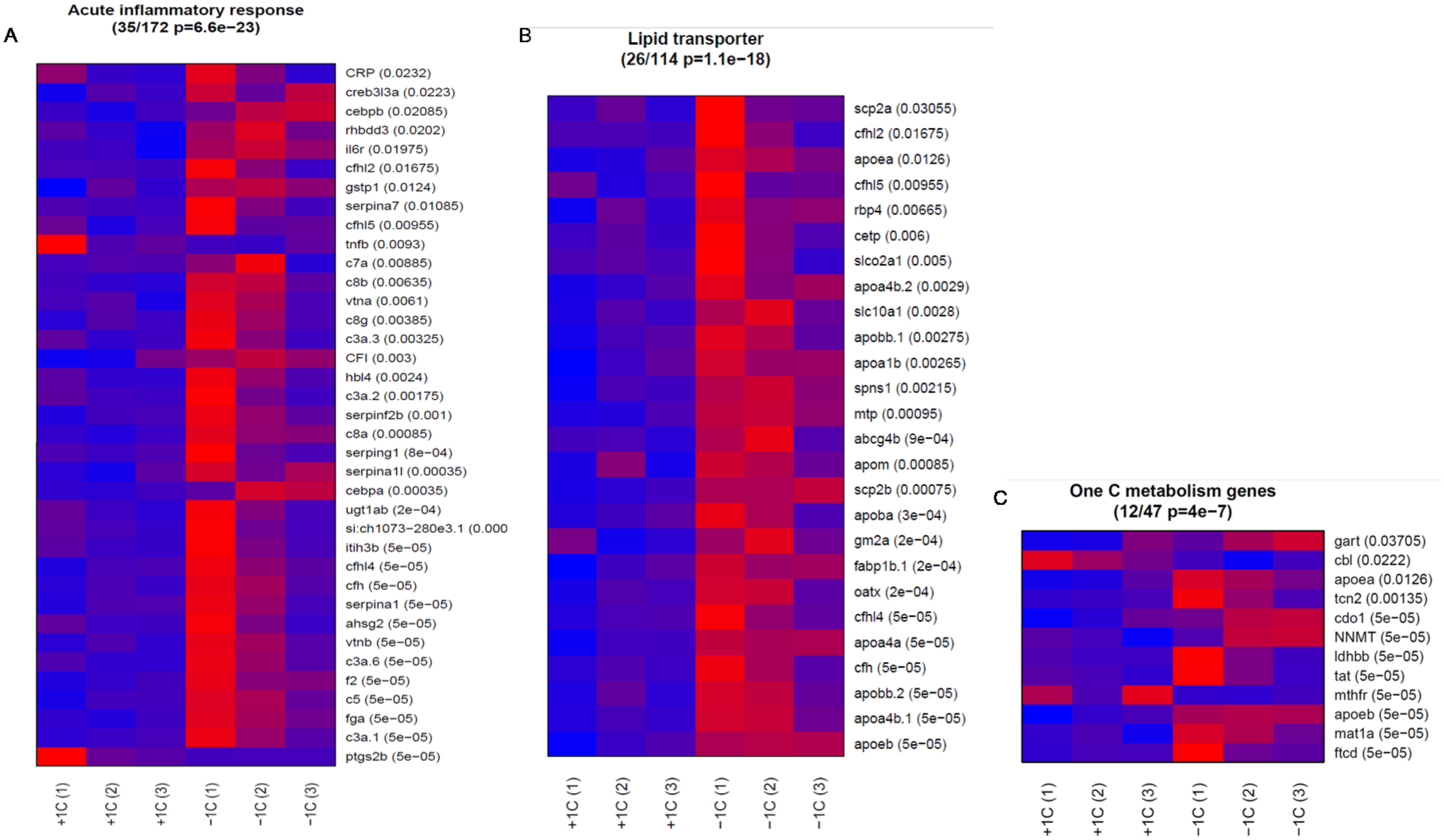


**Figure S3: Gene expression of significant GO terms affected from control (+1C) and low 1-C (-1C) $F_1$ embryos. A:** 35 significantly affected transcripts from the Acute inflammatory response GO term, **B:** 26 significantly affected transcripts from the Lipid transporter GO term **C:** 12 significantly affected transcripts from the 1-C metabolism genes. Expression levels are indicated by colour codes, with blue to red indicating min to max expression level for each gene, respectively. Each row of the heat map with its given gene name ID displays gene expression level of differentially expressed genes. P-values are indicated next to the gene names.

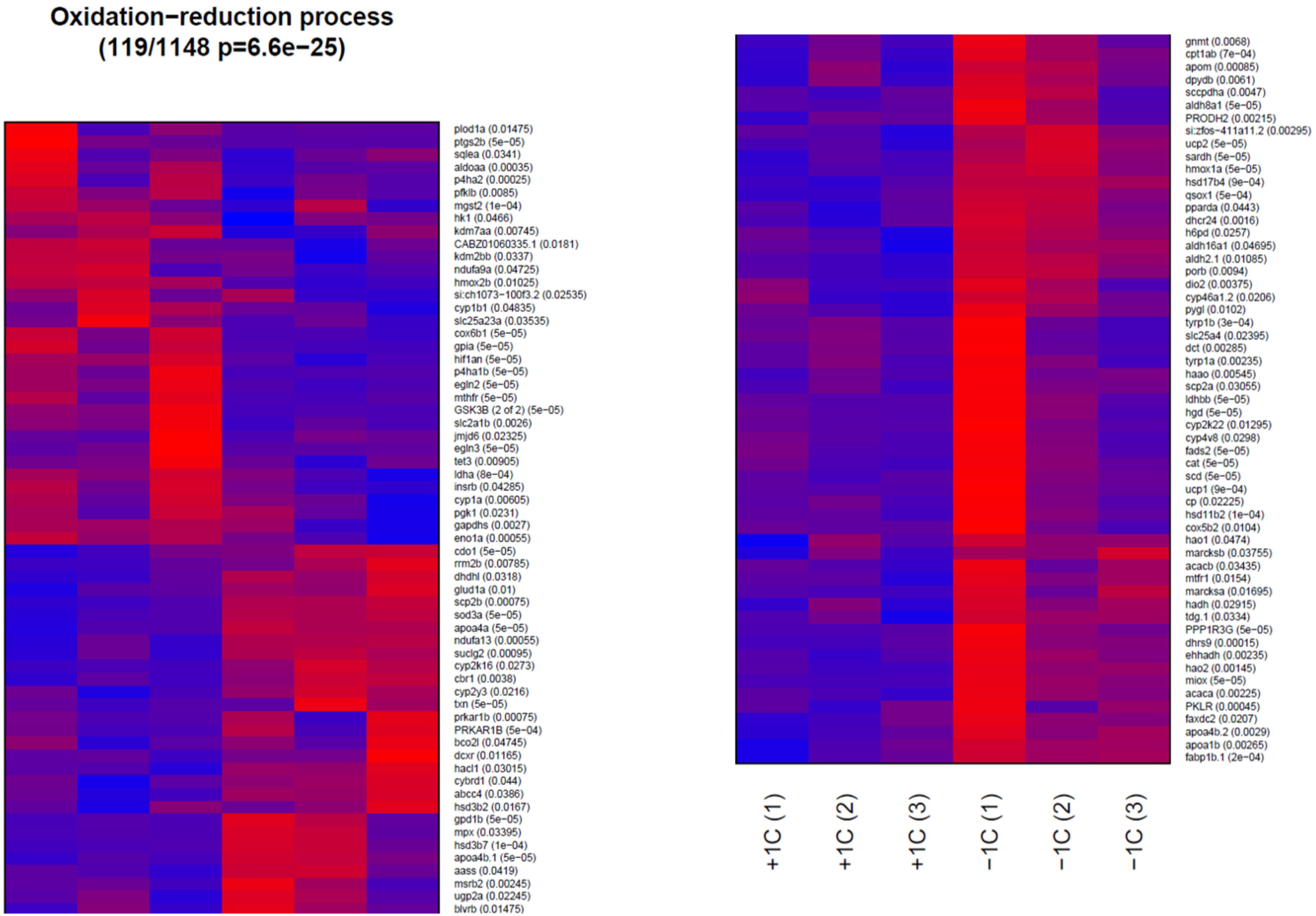


**Figure S4: Significantly affected transcripts in low 1-C embryos compared to control embryos from the oxidation-reduction process GO term.** In total 119 transcripts were differentially expressed in control (+1C) and low 1-C (-1C) $F_1$ embryos. Expression levels are indicated by colour codes, with blue to red indicating min to max expression level for each gene, respectively. Each row of the heat map with its given gene name ID displays gene expression level of differentially expressed genes. P-values are indicated next to the gene names.

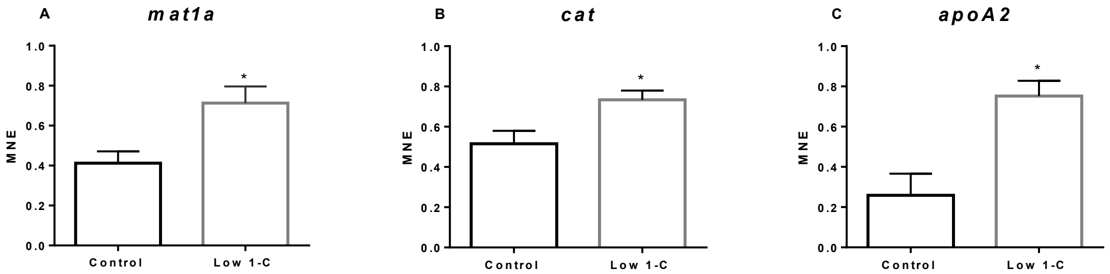


**Figure S5: RNA sequencing verification by RT-qPCR.** mRNA levels presented as mean normalized expression (MNE) in control embryos versus low 1-C embryos. MNE of target genes are normalized against *ef1a* and *tuba1* as reference genes. All data points presented are the means ± SD of 3 independent groups (egg incubation groups originating from independent parents from different feeding tanks) of 30 embryos at 26 somite stage. Significant differences are marked by asterisk (* = $p < 0.05$). Gene abbreviations, name and accession number are given in table S2.

## Supplementary tables:

**Table S1:** Standard procedures for zebrafish mating, handling and feeding used for both $F_0$ and $F_1$ generations.

| **Days post fertilization** | **Incubation devise** | **Incubation medium** | **Number of individuals** | **Special handling** | **Feed** | | | |
|---|---|---|---|---|---|---|---|---|
| | | | | | Artemia concentrate (µL) | | Gemma Micro 70 (mg) | |
| | | | | | morning | noon | morning | afternoon |
| 0-2.5 | Petri dish (80mm)* | E3 ** | 120 | Rinse eggs in E3, remove dead eggs | | | | |
| 2.5 - 4 | Petri dish (80mm)* | E3 | 120 | Change 80% of the water every day | | | | |
| 4 - 5 | Beakers 250 (mL)* | 100mL E3 | 60 | Transfer to beakers | | | | |
| 5 - 7 | Beakers 250 (mL)* | 100mL E3 | 60 | Start feeding Gemma Micro | | | 10 | 10 |
| 7 - 10 | Beakers 250 (mL)* | 200mL E3 | 60 | Start feeding artemia | 100 | | 10 | 10 |
| 10 - 15 | 1.5L Aquatic Habitat tanks | 0.5L E3 + 0.5L System water | 60 | Transfer to system tanks. Add additional 0.5L system water/day | 400 | 400 | 40 | 40 |
| 15 - 27 | 3L Aquatic Habitat tanks | System water | 60 | Turn on system circulation, low speed. Gradually increase circulation speed each day | 600 | 600 | 60 | 60 |
| 27 | 3L Aquatic Habitat tanks | System water | 60 | Start feeding experimental diets | Experimental diets: Control and Low 1-C | | | |

* The embryos and larvae were kept in an incubator at 28.5°C and 12/12h light and dark cycles
** E3 medium: https://wiki.zfin.org/display/prot

**Table S2:** Gene abbreviation, gene name, gene bank accession number, forward and reverse primer sequence, primer amplicon size and qRT-PCR efficiency for selected reference and target genes analysed.

| Gene abbr. | Gene name | Accession no. | Forward primer (5' - 3') | Reverse primer (5' - 3') | Amplicon size (bp) | PCR eff. |
|---|---|---|---|---|---|---|
| **Reference genes:** | | | | | | |
| *ef1α* | *Elongation factor 1 α* | NM_131263.1 | AGACAACCCCAAGGCTCTCA | CTCATGTCACGCACAGCAAA | 126 | 1,99 |
| *tuba1* | *tubulin, alpha 1c* | NM_001105126 | GGTGCCCTCAATGTGGATCT | GCCACAGAGAGCTGCTCATG | 131 | 2,06 |
| **Target genes:** | | | | | | |
| *mat1a* | *methionine adenosyltransferase 1, alpha* | NM_199871 | CGGCTCATCGGAAAAAACAG | GACCATAGCAGGCGGTGTTC | 131 | 2,12 |
| *cat* | *catalase* | NM_130912 | ACTACCAGTCAACTGCCCGTA | GAAGAACGTGCGCACTTGGG | 226 | 1,99 |
| *apoA2* | *apolipoprotein A-II* | NM_001130586 | TTCTTGCACTCCAAGTGTCAGT | CCCTCTAGTGCTGGCTCAAC | 131 | 2,01 |

**Table S3:** Main pathway and sub pathway enrichment analysis showing metabolites affected by dietary 1-C deficiency in $F_0$.

| Main Pathway Name | Main Pathway Enrichment (K*/M*)/(n/N) | Sub Pathway Name | Compounds In Pathway | | Sub Pathway Enrichment (k/m)/(n/N) | Statistically significant (p<0.05) metabolites affected (fold changes of Low 1-C / control). q-values (bold metabolite name indicate q-value < 0.1, others < 0.3) |
|---|---|---|---|---|---|---|
| | | | Significant (k) | Detected (m) | | |
| Amino acid | 2,39 | Alanine and Aspartate Metabolism | 2 | 6 | 3,9 | aspartate (-0.85), N-acetylasparagine (-0.82) |
| | | Glycine, Serine and Threonine Metabolism | 4 | 9 | 5,2 | **betaine** (-0.80), serine (+1.25) glycine (+1.34), |
| | | Tryptophan Metabolism | 2 | 6 | 3,9 | 5-hydroxyindoleacetate (-0.54), C-glycosyltryptophan (-0.89) |
| | | Creatine Metabolism | 1 | 4 | 2,9 | **guanidinoacetate** (+2.41) |
| | | Phenylalanine and Tyrosine Metabolism | 3 | 13 | 2,7 | N-acetylphenylalanine (+1.30), **N-acetyltyrosine** (+1.84), **3-methoxytyrosine** (+2.87) |
| | | Leucine, Isoleucine and Valine Metabolism | 4 | 21 | 2,2 | **N-acetylleucine** (+1.48), N-acetylisoleucine (+1.36), N-acetylvaline (+1.69), alpha-hydroxyisovalerate (-0.76) |
| | | Glutamate Metabolism | 2 | 11 | 2,1 | gamma-aminobutyrate (GABA) (-0.91), **carboxyethyl-GABA** (-0.81) |
| | | Urea cycle; Arginine and Proline Metabolism | 3 | 17 | 2,1 | ornithine (+2.07), citrulline (+1.29), **N-delta-acetylornithine** (+1.58) |
| | | Methionine, Cysteine, SAM, Taurine Metabolism | 3 | 18 | 2,0 | **methionine sulfone** (+2.31), **cystathionine** (+2.27), 2-hydroxybutyrate (+1.16) |
| | | Histidine Metabolism | 2 | 13 | 1,8 | 1-methylhistidine (+1.19), **imidazole lactate** (+1.96) |
| | | Lysine Metabolism | 2 | 13 | 1,8 | **2-aminoadipate** (+2.36), 6-oxopiperidine-2-carboxylic acid (+1.36) |
| | | Glutathione Metabolism | 1 | 8 | 1,5 | glutathione, reduced (GSH) (-0.69) |
| Vitamins | 1,67 | Vitamin B6 Metabolism | 4 | 4 | 11,8 | **pyridoxamine** (-0.39), **pyridoxamine phosphate** (-0.51), **pyridoxal** (-0.36), pyridoxate (-0.34) |
| Nucleotide | 0,61 | Pyrimidine Metabolism, Thymine containing | 1 | 5 | 2,4 | 3-aminoisobutyrate (+1.43) |
| | | Purine Metabolism, Xanthine/Inosine containing | 1 | 11 | 1,1 | xanthosine (+1.35) |
| | | Pyrimidine Metabolism, Uracil containing | 1 | 11 | 1,1 | **beta-alanine** (+2.06) |
| Lipid | 0,54 | Fatty Acid Metabolism (Acyl Choline) | 1 | 1 | 11,8 | palmitoylcholine (-0.72) |
| | | Endocannabinoid | 3 | 8 | 4,4 | oleoyl ethanolamide (+1.67), stearoyl ethanolamide (+1.50), **linoleoyl ethanolamide** (+2.36) |
| | | Fatty Acid, Dicarboxylate | 2 | 11 | 2,1 | maleate (-0.76), **undecanedioate** (-0.59) |
| | | Fatty Acid Metabolism(Acyl Carnitine) | 1 | 13 | 0,9 | myristoleoylcarnitine (-0.57) |
| | | Sphingolipid Metabolism | 1 | 22 | 0,5 | palmitoyl dihydrosphingomyelin, d18:0/16:0 (-0.75) |
| | | Lysolipid | 1 | 30 | 0,4 | 1-linoleoyl-GPA ,18:2 (+1.40) |
| | | Phospholipid Metabolism | 1 | 36 | 0,3 | choline phosphate (-0.85) |
| Peptide | 0,48 | Gamma-glutamyl Amino Acid | 1 | 12 | 1,0 | gamma-glutamylglycine (+1.60) |
| Carbohydrate | 0,26 | Nucleotide Sugar | 1 | 6 | 2,0 | UDP-N-acetylglucosamine (-0.86) |

48

**N**: total number of detected metabolites (566), **n**: total number of metabolites affected by the diet (48), **M**: number of detected metabolites of main pathway, **m**: number of detected metabolites of sub pathway, **K**: number of metabolites affected by diet in main pathway, **k**: number of metabolites affected by diet in sub pathway. The diet effect is indicated as fold changes, positive and negative values indicate higher and lower intensity in low 1-C group than control, respectively (log2(low 1-C/ctl)). Q-values < 0.1 are indicated with bold metabolite name.

**Table S4:** Complete list of 364 differentially expressed genes in low 1-C embryos compared to control embryos.
*Attached separately as excel file*

| gene_id | gene | locus | sample_1 | sample_2 | status | value_1 | value_2 | log2.fold_change. | test_stat | p_value | q_value | significant |
|---|---|---|---|---|---|---|---|---|---|---|---|---|
| **ENSDARG00000094023** | **si:dkeyp-79f4.1** | **4:23401768-23402535** | **low1C** | **ctl** | **OK** | **0,536847** | **0** | **#NAVN?** | **NA** | **0,00015** | **0,01096** | **yes** |
| **ENSDARG00000082452** | **U4** | **17:23328304-23328445** | **low1C** | **ctl** | **OK** | **0** | **26,0438** | **Inf** | **NA** | **0,00005** | **0,00421538** | **yes** |
| **ENSDARG00000098549** | **U6** | **4:62994355-62994462** | **low1C** | **ctl** | **OK** | **0** | **60,9421** | **Inf** | **NA** | **0,00005** | **0,00421538** | **yes** |
| **ENSDARG00000083431** | **U4** | **25:5213091-5213232** | **low1C** | **ctl** | **OK** | **0** | **15,1922** | **Inf** | **NA** | **0,00095** | **0,0464045** | **yes** |
| **ENSDARG00000062788** | **irg1l** | **14:11121037-11124596** | **low1C** | **ctl** | **OK** | **1,34071** | **18,992** | **3,82432** | **4,89773** | **0,00005** | **0,00421538** | **yes** |
| **ENSDARG00000052468** | **GSK3B (2 of 2)** | **1:48726128-48765129** | **low1C** | **ctl** | **OK** | **0,54925** | **6,25321** | **3,50906** | **2,73127** | **0,00005** | **0,00421538** | **yes** |
| **ENSDARG00000081108** | **U5** | **20:48779134-48779250** | **low1C** | **ctl** | **OK** | **113,134** | **1105,32** | **3,28836** | **4,57588** | **0,00005** | **0,00421538** | **yes** |
| **ENSDARG00000104919** | **si:ch211-153b23.3** | **14:11124781-11132075** | **low1C** | **ctl** | **OK** | **0,232334** | **2,2227** | **3,25804** | **5,27163** | **0,00005** | **0,00421538** | **yes** |
| **ENSDARG00000032553** | **egln3** | **17:9755991-9780871** | **low1C** | **ctl** | **OK** | **2,98372** | **21,5616** | **2,85328** | **5,85144** | **0,00005** | **0,00421538** | **yes** |
| **ENSDARG00000082424** | **U4** | **17:23315576-23315717** | **low1C** | **ctl** | **OK** | **38,3838** | **258,38** | **2,75092** | **3,38882** | **0,0001** | **0,00772511** | **yes** |
| **ENSDARG00000100756** | **U1** | **3:9152925-9153074** | **low1C** | **ctl** | **OK** | **21,8371** | **130,994** | **2,58465** | **2,7727** | **0,0009** | **0,0447093** | **yes** |
| **ENSDARG00000075339** | **ipp** | **2:3152162-3180676** | **low1C** | **ctl** | **OK** | **2,00597** | **11,7599** | **2,5515** | **4,84989** | **0,00005** | **0,00421538** | **yes** |

| | | | | | | | | | | | | |
|---|---|---|---|---|---|---|---|---|---|---|---|---|
| ENSDARG00000101135 | si:dkey-85k7.7 | 7:21592998-21608008 | low1C | ctl | OK | 2,0592 | 11,9117 | 2,53223 | 6,75768 | 0,00005 | 0,00421538 | yes |
| ENSDARG00000103481 | Metazoa_SRP | 11:12239840-12240137 | low1C | ctl | OK | 2,59553 | 14,9127 | 2,52244 | 2,81522 | 0,00025 | 0,0166061 | yes |
| ENSDARG00000062562 | egln2 | 15:8359528-8406926 | low1C | ctl | OK | 0,207955 | 1,18353 | 2,50876 | 4,10033 | 0,00005 | 0,00421538 | yes |
| ENSDARG00000084991 | SNORA62 | 3:6151961-6152097 | low1C | ctl | OK | 32,3272 | 182,879 | 2,50007 | 2,64479 | 0,00065 | 0,0365333 | yes |
| ENSDARG00000103556 | si:ch211-218g23.6 | 11:29487411-29489914 | low1C | ctl | OK | 0,357233 | 1,9715 | 2,46436 | 3,4497 | 0,00005 | 0,00421538 | yes |
| ENSDARG00000081192 | U5 | 20:23333589-23333704 | low1C | ctl | OK | 2693,83 | 14588,8 | 2,43713 | 7,36884 | 0,00005 | 0,00421538 | yes |
| ENSDARG00000077169 | si:ch211-153b23.4 | 14:11144483-11150710 | low1C | ctl | OK | 0,441944 | 2,35856 | 2,41597 | 4,6661 | 0,00005 | 0,00421538 | yes |
| ENSDARG00000105097 | Metazoa_SRP | 11:11400211-11400509 | low1C | ctl | OK | 4,65037 | 23,8692 | 2,35974 | 3,34461 | 0,00005 | 0,00421538 | yes |
| ENSDARG00000101489 | U1 | 4:41438317-41438482 | low1C | ctl | OK | 20,5643 | 104,113 | 2,33993 | 2,79481 | 0,0005 | 0,0292267 | yes |
| ENSDARG00000102181 | Metazoa_SRP | 11:11477184-11477481 | low1C | ctl | OK | 2,07019 | 10,4767 | 2,33935 | 2,64741 | 0,0009 | 0,0447093 | yes |
| ENSDARG00000091996 | si:ch211-117m20.5 | 5:30014378-30015748 | low1C | ctl | OK | 0,741185 | 3,67089 | 2,30823 | 3,14501 | 0,00005 | 0,00421538 | yes |
| ENSDARG00000089617 | RNaseP_nuc | 2:37844278-37844581 | low1C | ctl | OK | 29,3689 | 145,299 | 2,30666 | 5,40585 | 0,00005 | 0,00421538 | yes |
| ENSDARG00000086105 | Vault | 14:2489384-2489489 | low1C | ctl | OK | 2410,5 | 11863,1 | 2,29907 | 6,16995 | 0,00005 | 0,00421538 | yes |
| ENSDARG00000085497 | RNaseP_nuc | 2:42737319-42737617 | low1C | ctl | OK | 17,2493 | 82,4628 | 2,25721 | 4,54862 | 0,00005 | 0,00421538 | yes |
| ENSDARG00000034211 | capn2l | 22:26617034-26646980 | low1C | ctl | OK | 0,221269 | 1,05428 | 2,25238 | 3,28174 | 0,00005 | 0,00421538 | yes |

| | | | | | | | | | | | | |
|---|---|---|---|---|---|---|---|---|---|---|---|---|
| ENSDARG00000098933 | Metazoa_SRP | 11:12245901-12632880 | low1C | ctl | OK | 14,7642 | 67,6417 | 2,19581 | 3,36491 | 0,00005 | 0,00421538 | yes |
| ENSDARG00000089313 | U4 | 4:14728262-14737845 | low1C | ctl | OK | 1470,95 | 6735,93 | 2,19513 | 3,02495 | 0,00025 | 0,0166061 | yes |
| ENSDARG00000056376 | ankrd37 | 1:17002488-17005836 | low1C | ctl | OK | 3,15399 | 14,3074 | 2,18151 | 5,00969 | 0,00005 | 0,00421538 | yes |
| ENSDARG00000081029 | U5 | 20:48781039-48781153 | low1C | ctl | OK | 184,39 | 833,857 | 2,17704 | 3,31457 | 0,00005 | 0,00421538 | yes |
| ENSDARG00000099971 | 5S_rRNA | KN150576.1:8255-8374 | low1C | ctl | OK | 1199,21 | 5292,74 | 2,14193 | 5,47411 | 0,00005 | 0,00421538 | yes |
| ENSDARG00000070286 | si:ch211-113a14.19 | 25:35791636-35792023 | low1C | ctl | OK | 3,99176 | 17,4909 | 2,1315 | 3,45558 | 0,00005 | 0,00421538 | yes |
| ENSDARG00000103491 | Metazoa_SRP | 11:11463567-11463864 | low1C | ctl | OK | 16,1807 | 70,7268 | 2,12799 | 4,21956 | 0,00005 | 0,00421538 | yes |
| ENSDARG00000090149 | HIST4H4 (4 of 5) | 25:34540844-34541156 | low1C | ctl | OK | 4,44299 | 18,8394 | 2,08415 | 2,88508 | 0,00015 | 0,01096 | yes |
| ENSDARG00000080562 | U6atac | 21:6287400-6287528 | low1C | ctl | OK | 200,252 | 839,673 | 2,06801 | 3,68353 | 0,00005 | 0,00421538 | yes |
| ENSDARG00000090273 | Vault | 14:2488593-2488721 | low1C | ctl | OK | 2159,99 | 9042,69 | 2,06572 | 3,88504 | 0,00005 | 0,00421538 | yes |
| ENSDARG00000087236 | HIST1H4B | 25:35812927-35813239 | low1C | ctl | OK | 5,41246 | 22,1767 | 2,03469 | 3,12266 | 0,00005 | 0,00421538 | yes |
| ENSDARG00000080410 | U11 | 19:16157463-16157599 | low1C | ctl | OK | 231,533 | 929,494 | 2,00523 | 4,0563 | 0,00005 | 0,00421538 | yes |
| ENSDARG00000087732 | Metazoa_SRP | 3:30117896-30118194 | low1C | ctl | OK | 1035,43 | 4095,31 | 1,98374 | 7,20521 | 0,00005 | 0,00421538 | yes |
| ENSDARG00000010276 | ptgs2b | 20:34218938-34224536 | low1C | ctl | OK | 1,04185 | 4,01465 | 1,94612 | 4,44793 | 0,00005 | 0,00421538 | yes |
| ENSDARG00000101840 | U3 | 1:15765587-15765803 | low1C | ctl | OK | 654,764 | 2486,55 | 1,9251 | 6,78776 | 0,00005 | 0,00421538 | yes |

| | | | | | | | | | | | | |
|---|---|---|---|---|---|---|---|---|---|---|---|---|
| ENSDARG00000090182 | si:dkey-261m9.18 | 25:34639275-34639662 | low1C | ctl | OK | 3,11268 | 11,7947 | 1,92191 | 2,84298 | 0,00005 | 0,00421538 | yes |
| ENSDARG00000101384 | Metazoa_SRP | 11:11454269-11454566 | low1C | ctl | OK | 7,99724 | 29,9953 | 1,90716 | 3,08955 | 0,00005 | 0,00421538 | yes |
| ENSDARG00000084089 | Vault | 15:21316458-21316550 | low1C | ctl | OK | 387,185 | 1450,22 | 1,90518 | 2,69702 | 0,0002 | 0,0139175 | yes |
| ENSDARG00000081270 | rn7sk | 13:695612-695934 | low1C | ctl | OK | 436,011 | 1627,37 | 1,9001 | 7,13452 | 0,00005 | 0,00421538 | yes |
| ENSDARG00000087472 | HIST4H4 (2 of 5) | 25:35776073-35776385 | low1C | ctl | OK | 12,3969 | 45,6364 | 1,88021 | 3,62679 | 0,00005 | 0,00421538 | yes |
| ENSDARG00000045230 | cox6b1 | 24:39718205-39722738 | low1C | ctl | OK | 14,6555 | 53,917 | 1,8793 | 5,61137 | 0,00005 | 0,00421538 | yes |
| ENSDARG00000100545 | Metazoa_SRP | 11:12184380-12184677 | low1C | ctl | OK | 8,58364 | 31,0703 | 1,85588 | 3,02921 | 0,00005 | 0,00421538 | yes |
| ENSDARG00000082538 | U5 | 20:16528206-16528322 | low1C | ctl | OK | 108,448 | 390,588 | 1,84864 | 2,3808 | 0,0004 | 0,0243556 | yes |
| ENSDARG00000082628 | U1 | 10:211781-211947 | low1C | ctl | OK | 68,1832 | 243,43 | 1,83602 | 3,22143 | 0,00005 | 0,00421538 | yes |
| ENSDARG00000084628 | SNORA27 | 20:23182559-23182696 | low1C | ctl | OK | 175,713 | 620,119 | 1,81932 | 3,40924 | 0,00005 | 0,00421538 | yes |
| ENSDARG00000089044 | HIST4H4 (3 of 5) | 25:35783062-35783374 | low1C | ctl | OK | 6,94645 | 24,3983 | 1,81243 | 2,9653 | 0,00005 | 0,00421538 | yes |
| ENSDARG00000080685 | SNORA50 | 3:55224901-55225036 | low1C | ctl | OK | 138,163 | 484,232 | 1,80932 | 3,05992 | 0,00005 | 0,00421538 | yes |
| ENSDARG00000099668 | snopsi28S-3327 | 19:16124093-16124240 | low1C | ctl | OK | 198,577 | 641,406 | 1,69154 | 3,45066 | 0,00005 | 0,00421538 | yes |
| ENSDARG00000087543 | si:dkey-108k21.12 | 25:34551646-34552140 | low1C | ctl | OK | 8,61012 | 27,2833 | 1,66392 | 2,69346 | 0,00005 | 0,00421538 | yes |
| ENSDARG00000089823 | si:ch211-113a14.6 | 25:35760530-35760842 | low1C | ctl | OK | 5,80862 | 17,9307 | 1,62617 | 2,46954 | 0,0004 | 0,0243556 | yes |

| | | | | | | | | | | | | |
|---|---|---|---|---|---|---|---|---|---|---|---|---|
| ENSDARG00000101500 | Metazoa_SRP | 11:12225287-12225584 | low1C | ctl | OK | 17,0547 | 51,7144 | 1,6004 | 3,16945 | 0,00005 | 0,00421538 | yes |
| ENSDARG00000081931 | SNORA16 | 19:16124945-16125081 | low1C | ctl | OK | 1095,45 | 3311,51 | 1,59597 | 4,672 | 0,00005 | 0,00421538 | yes |
| ENSDARG00000071082 | p4ha1b | 17:20127566-20147370 | low1C | ctl | OK | 13,1177 | 39,5702 | 1,5929 | 6,06858 | 0,00005 | 0,00421538 | yes |
| ENSDARG00000086604 | si:ch211-113a14.25 | 25:35817417-35817804 | low1C | ctl | OK | 4,97397 | 14,7158 | 1,5649 | 2,63452 | 0,00005 | 0,00421538 | yes |
| ENSDARG00000093071 | zgc:110434 | 7:6226221-6226608 | low1C | ctl | OK | 5,05388 | 14,8688 | 1,55682 | 2,60214 | 0,00005 | 0,00421538 | yes |
| ENSDARG00000091205 | si:ch211-113a14.19 | 7:73632521-73632909 | low1C | ctl | OK | 3,09419 | 8,97424 | 1,53623 | 2,26509 | 0,0007 | 0,0382405 | yes |
| ENSDARG00000099271 | 5_8S_rRNA | KN149861.1:7180-7336 | low1C | ctl | OK | 293,29 | 850,627 | 1,5362 | 3,47484 | 0,00005 | 0,00421538 | yes |
| ENSDARG00000051775 | si:dkey-108k21.15 | 25:34559683-34560070 | low1C | ctl | OK | 10,0485 | 28,7906 | 1,51862 | 3,0866 | 0,00005 | 0,00421538 | yes |
| ENSDARG00000085485 | SNORA26 | 20:23182968-23183090 | low1C | ctl | OK | 306,641 | 875,31 | 1,51324 | 2,8596 | 0,00005 | 0,00421538 | yes |
| ENSDARG00000070265 | si:dkey-261m9.7 | 25:34628445-34628832 | low1C | ctl | OK | 10,4697 | 29,6372 | 1,50118 | 3,11221 | 0,00005 | 0,00421538 | yes |
| ENSDARG00000086552 | zgc:154164 | 7:6231967-6232548 | low1C | ctl | OK | 12,4671 | 34,9665 | 1,48785 | 3,24355 | 0,00005 | 0,00421538 | yes |
| ENSDARG00000090591 | si:dkey-261m9.13 | 25:34638090-34638402 | low1C | ctl | OK | 11,9338 | 33,3894 | 1,48434 | 2,78264 | 0,00005 | 0,00421538 | yes |
| ENSDARG00000101249 | HIST1H4J (2 of 2) | 7:5837576-5838171 | low1C | ctl | OK | 2,36026 | 6,50212 | 1,46197 | 2,42055 | 0,0003 | 0,0193412 | yes |
| ENSDARG00000104706 | U3 | 7:7545593-7545807 | low1C | ctl | OK | 590,628 | 1624,18 | 1,45939 | 3,86776 | 0,00005 | 0,00421538 | yes |
| ENSDARG00000075379 | si:dkey-261m9.11 | 25:34635401-34635788 | low1C | ctl | OK | 7,46727 | 20,1941 | 1,43528 | 2,72679 | 0,00005 | 0,00421538 | yes |

| | | | | | | | | | | | | |
|---|---|---|---|---|---|---|---|---|---|---|---|---|
| ENSDARG00000102435 | plekhf1 | 7:45703170-45704590 | low1C | ctl | OK | 3,19026 | 8,61876 | 1,43381 | 3,40587 | 0,00005 | 0,00421538 | yes |
| ENSDARG00000089719 | si:dkey-108k21.11 | 25:34549261-34549710 | low1C | ctl | OK | 10,6629 | 28,6823 | 1,42757 | 3,23291 | 0,00005 | 0,00421538 | yes |
| ENSDARG00000097103 | zgc:165555 | 25:34560843-34561155 | low1C | ctl | OK | 9,00982 | 23,993 | 1,41304 | 2,40268 | 0,0003 | 0,0193412 | yes |
| ENSDARG00000095340 | si:ch73-36p18.2 | 25:35762739-35763150 | low1C | ctl | OK | 8,3795 | 22,2038 | 1,40587 | 2,83483 | 0,00005 | 0,00421538 | yes |
| ENSDARG00000051732 | si:ch211-113a14.7 | 25:35761613-35762000 | low1C | ctl | OK | 7,49166 | 19,8218 | 1,40373 | 2,6321 | 0,00005 | 0,00421538 | yes |
| ENSDARG00000099123 | zgc:110434 | 7:5835310-5835953 | low1C | ctl | OK | 5,70632 | 15,0619 | 1,40027 | 3,1118 | 0,00005 | 0,00421538 | yes |
| ENSDARG00000105169 | SCARNA14 | 15:43993989-43994120 | low1C | ctl | OK | 184,368 | 476,807 | 1,37082 | 2,34731 | 0,00015 | 0,01096 | yes |
| ENSDARG00000100279 | CU457819.2 | 7:6218466-6219610 | low1C | ctl | OK | 9,53139 | 24,6039 | 1,36813 | 2,71732 | 0,00005 | 0,00421538 | yes |
| ENSDARG00000075220 | si:ch211-113a14.19 | 7:73600357-73600745 | low1C | ctl | OK | 12,224 | 31,0342 | 1,34414 | 2,89384 | 0,00005 | 0,00421538 | yes |
| ENSDARG00000102172 | si:ch73-36p18.2 | 7:6231080-6231491 | low1C | ctl | OK | 7,79783 | 19,7418 | 1,34011 | 2,59993 | 0,00005 | 0,00421538 | yes |
| ENSDARG00000102011 | Metazoa_SRP | 11:12179130-12179427 | low1C | ctl | OK | 25,4629 | 64,428 | 1,33929 | 2,95085 | 0,00005 | 0,00421538 | yes |
| ENSDARG00000097020 | si:dkey-261m9.8 | 25:34629434-34629845 | low1C | ctl | OK | 11,1079 | 27,7675 | 1,32181 | 2,89169 | 0,00005 | 0,00421538 | yes |
| ENSDARG00000068928 | si:dkey-108k21.26 | 25:34605350-34605737 | low1C | ctl | OK | 9,01278 | 22,5166 | 1,32095 | 2,60838 | 0,00005 | 0,00421538 | yes |
| ENSDARG00000104115 | 5_8S_rRNA | 1:3784199-3784354 | low1C | ctl | OK | 156,251 | 388,287 | 1,31326 | 2,55504 | 0,00005 | 0,00421538 | yes |
| ENSDARG00000070287 | zgc:173552 | 25:35799854-35800265 | low1C | ctl | OK | 5,66969 | 14,0824 | 1,31256 | 2,34509 | 0,00025 | 0,0166061 | yes |

| | | | | | | | | | | | | |
|---|---|---|---|---|---|---|---|---|---|---|---|---|
| ENSDARG00000015543 | s100a1 | 16:29450941-29452592 | low1C | ctl | OK | 1,87139 | 4,63992 | 1,30999 | 2,11546 | 0,001 | 0,0481758 | yes |
| ENSDARG00000082850 | U8 | 10:22877819-22877952 | low1C | ctl | OK | 201,231 | 491,11 | 1,28719 | 2,27151 | 0,0004 | 0,0243556 | yes |
| ENSDARG00000041294 | noxo1a | 3:61924549-61931972 | low1C | ctl | OK | 4,66896 | 11,3638 | 1,28327 | 4,20379 | 0,00005 | 0,00421538 | yes |
| ENSDARG00000071596 | neto1l | 24:15753297-15992150 | low1C | ctl | OK | 0,452294 | 1,09487 | 1,27543 | 2,43585 | 0,00005 | 0,00421538 | yes |
| ENSDARG00000074260 | si:ch211-113a14.25 | 7:73623443-73623831 | low1C | ctl | OK | 14,8161 | 35,6856 | 1,26818 | 2,8869 | 0,00005 | 0,00421538 | yes |
| ENSDARG00000102946 | si:ch73-167f10.1 | 3:45233846-45237689 | low1C | ctl | OK | 5,67751 | 13,659 | 1,26652 | 2,20896 | 0,00045 | 0,0270247 | yes |
| ENSDARG00000051734 | si:ch211-113a14.16 | 25:35787200-35787587 | low1C | ctl | OK | 9,49585 | 22,8395 | 1,26616 | 2,52214 | 0,00005 | 0,00421538 | yes |
| ENSDARG00000056108 | ndufa4 | 12:9842649-9846483 | low1C | ctl | OK | 6,74401 | 16,1869 | 1,26314 | 3,89467 | 0,00005 | 0,00421538 | yes |
| ENSDARG00000075730 | dnph1 | 13:4089832-4095459 | low1C | ctl | OK | 4,88463 | 11,6568 | 1,25485 | 2,71079 | 0,00005 | 0,00421538 | yes |
| ENSDARG00000051736 | zgc:114037 | 25:35778804-35779286 | low1C | ctl | OK | 5,91732 | 13,9568 | 1,23795 | 2,43127 | 0,00015 | 0,01096 | yes |
| ENSDARG00000078941 | wu:fe11b02 | 25:34650659-34677389 | low1C | ctl | OK | 6,00583 | 14,1558 | 1,23696 | 3,47238 | 0,00005 | 0,00421538 | yes |
| ENSDARG00000091382 | HIST4H4 (5 of 5) | 7:6221820-6222132 | low1C | ctl | OK | 17,0723 | 40,2245 | 1,23642 | 2,52053 | 0,00005 | 0,00421538 | yes |
| ENSDARG00000100906 | zgc:195633 | 7:5853546-5854332 | low1C | ctl | OK | 3,07081 | 7,19945 | 1,22927 | 2,46861 | 0,0001 | 0,00772511 | yes |
| ENSDARG00000087670 | si:dkey-108k21.28 | 25:34608621-34608933 | low1C | ctl | OK | 13,9269 | 32,3592 | 1,2163 | 2,34071 | 0,0001 | 0,00772511 | yes |
| ENSDARG00000103819 | si:ch73-36p18.2 | 7:5783461-5784061 | low1C | ctl | OK | 10,1737 | 23,2683 | 1,19352 | 2,97755 | 0,00005 | 0,00421538 | yes |

| | | | | | | | | | | | | |
|---|---|---|---|---|---|---|---|---|---|---|---|---|
| **ENSDARG00000102700** | **FP236157.1** | **1:58794190-58798723** | **low1C** | **ctl** | **OK** | **0,43555** | **0,98792** | **1,18156** | **2,43052** | **0,0002** | **0,0139175** | **yes** |
| **ENSDARG00000069135** | **ppp1r15a** | **7:71262904-71268268** | **low1C** | **ctl** | **OK** | **12,3907** | **28,0017** | **1,17625** | **4,00474** | **0,00005** | **0,00421538** | **yes** |
| **ENSDARG00000100903** | **Y_RNA** | **KN150170.1:35263-35370** | **low1C** | **ctl** | **OK** | **4224,59** | **9540,46** | **1,17525** | **3,59183** | **0,00005** | **0,00421538** | **yes** |
| **ENSDARG00000100210** | **wu:fe11b02** | **7:6220676-6221051** | **low1C** | **ctl** | **OK** | **22,0309** | **49,6546** | **1,1724** | **2,88849** | **0,00005** | **0,00421538** | **yes** |
| **ENSDARG00000096596** | **zgc:173552** | **25:35815847-35816258** | **low1C** | **ctl** | **OK** | **12,1467** | **27,3093** | **1,16883** | **2,57981** | **0,0001** | **0,00772511** | **yes** |
| **ENSDARG00000099920** | **si:ch211-113a14.6** | **7:5846577-5847340** | **low1C** | **ctl** | **OK** | **3,00732** | **6,7602** | **1,16859** | **2,26715** | **0,0001** | **0,00772511** | **yes** |
| **ENSDARG00000099351** | **igfbp1a** | **20:6818826-6822741** | **low1C** | **ctl** | **OK** | **7,33405** | **16,0646** | **1,1312** | **2,9172** | **0,00005** | **0,00421538** | **yes** |
| **ENSDARG00000055046** | **ponzr5** | **7:22386408-22392814** | **low1C** | **ctl** | **OK** | **5,5808** | **12,0298** | **1,10806** | **2,56128** | **0,00005** | **0,00421538** | **yes** |
| **ENSDARG00000051737** | **zgc:173552** | **25:35779903-35780314** | **low1C** | **ctl** | **OK** | **11,3391** | **24,2182** | **1,09478** | **2,3364** | **0,0001** | **0,00772511** | **yes** |
| **ENSDARG00000012987** | **gpia** | **25:35408674-35434057** | **low1C** | **ctl** | **OK** | **14,9885** | **31,8565** | **1,08773** | **3,15768** | **0,00005** | **0,00421538** | **yes** |
| **ENSDARG00000042492** | **si:dkey-250d21.1** | **10:32122494-32128929** | **low1C** | **ctl** | **OK** | **0,591321** | **1,25515** | **1,08584** | **1,95031** | **0,0009** | **0,0447093** | **yes** |
| **ENSDARG00000091446** | **HIST2H4A** | **25:34642428-34642740** | **low1C** | **ctl** | **OK** | **13,7404** | **29,1205** | **1,08361** | **2,05872** | **0,0007** | **0,0382405** | **yes** |
| **ENSDARG00000083480** | **AC024175.23** | **MT:950-1019** | **low1C** | **ctl** | **OK** | **4803,59** | **10173,8** | **1,08267** | **1,98398** | **0,00085** | **0,0434566** | **yes** |
| **ENSDARG00000086304** | **si:ch73-36p18.5** | **25:34622272-34622659** | **low1C** | **ctl** | **OK** | **12,8681** | **27,2514** | **1,08254** | **2,31597** | **0,00005** | **0,00421538** | **yes** |
| **ENSDARG00000021343** | **wsb1** | **15:31281041-31296583** | **low1C** | **ctl** | **OK** | **24,0955** | **50,5673** | **1,06944** | **3,22542** | **0,00005** | **0,00421538** | **yes** |

| | | | | | | | | | | | | |
|---|---|---|---|---|---|---|---|---|---|---|---|---|
| ENSDARG00000058508 | CFAP70 | 3:12036960-12087556 | low1C | ctl | OK | 1,46114 | 3,05588 | 1,06449 | 2,70582 | 0,00005 | 0,00421538 | yes |
| ENSDARG00000086555 | HIST4H4 (1 of 5) | 7:73634439-73634751 | low1C | ctl | OK | 18,5025 | 38,4772 | 1,05628 | 2,19787 | 0,0002 | 0,0139175 | yes |
| ENSDARG00000098875 | Metazoa_SRP | 11:12203684-12203981 | low1C | ctl | OK | 42,6125 | 88,6035 | 1,05609 | 2,62582 | 0,00005 | 0,00421538 | yes |
| ENSDARG00000082753 | AC024175.17 | MT:1019-1971 | low1C | ctl | OK | 1723,8 | 3583,48 | 1,05577 | 3,87819 | 0,00005 | 0,00421538 | yes |
| ENSDARG00000074937 | si:dkey-261m9.15 | 25:34646882-34647269 | low1C | ctl | OK | 9,6133 | 19,9572 | 1,0538 | 2,08189 | 0,0006 | 0,0340505 | yes |
| ENSDARG00000058206 | si:ch211-153b23.5 | 14:11151485-11154988 | low1C | ctl | OK | 14,5258 | 29,4497 | 1,01963 | 3,17676 | 0,00005 | 0,00421538 | yes |
| ENSDARG00000031915 | hif1an | 13:29822182-29849765 | low1C | ctl | OK | 13,1071 | 26,5358 | 1,0176 | 2,44416 | 0,00005 | 0,00421538 | yes |
| ENSDARG00000093195 | si:dkey-261m9.6 | 25:34627131-34627506 | low1C | ctl | OK | 45,3926 | 91,455 | 1,0106 | 2,97502 | 0,00005 | 0,00421538 | yes |
| ENSDARG00000097258 | si:dkey-108k21.25 | 25:34604167-34604578 | low1C | ctl | OK | 9,22917 | 18,4708 | 1,00097 | 1,99526 | 0,001 | 0,0481758 | yes |
| ENSDARG00000092395 | si:ch73-36p18.1 | 25:34616373-34616988 | low1C | ctl | OK | 8,94523 | 17,8513 | 0,996836 | 2,42614 | 0,00005 | 0,00421538 | yes |
| ENSDARG00000104944 | HIST2H3A (2 of 2) | 25:34615322-34615733 | low1C | ctl | OK | 11,0479 | 21,6484 | 0,970484 | 2,0692 | 0,00065 | 0,0365333 | yes |
| ENSDARG00000091770 | zgc:173552 | 7:73594850-73595261 | low1C | ctl | OK | 20,663 | 40,3906 | 0,966969 | 2,48827 | 0,00005 | 0,00421538 | yes |
| ENSDARG00000039547 | si:ch211-113a14.22 | 25:35797406-35797781 | low1C | ctl | OK | 40,8671 | 79,8683 | 0,966681 | 2,80739 | 0,00005 | 0,00421538 | yes |
| ENSDARG00000097785 | si:ch73-36p18.2 | 25:34634151-34634562 | low1C | ctl | OK | 18,5287 | 36,1953 | 0,966045 | 2,39505 | 0,00005 | 0,00421538 | yes |
| ENSDARG00000076930 | HIST1H4F | 25:34632121-34632433 | low1C | ctl | OK | 29,0031 | 56,1435 | 0,952913 | 2,22389 | 0,0002 | 0,0139175 | yes |

| | | | | | | | | | | | | |
|---|---|---|---|---|---|---|---|---|---|---|---|---|
| ENSDARG00000060113 | znf395a | 17:16082226-16100451 | low1C | ctl | OK | 34,2009 | 66,1609 | 0,951946 | 3,45632 | 0,00005 | 0,00421538 | yes |
| ENSDARG00000070280 | zgc:173652 | 25:35798681-35799293 | low1C | ctl | OK | 7,67183 | 14,6643 | 0,93466 | 2,13521 | 0,00025 | 0,0166061 | yes |
| ENSDARG00000042210 | sema3ab | 18:9354097-9470930 | low1C | ctl | OK | 1,04988 | 1,99696 | 0,927575 | 2,076 | 0,0007 | 0,0382405 | yes |
| ENSDARG00000031929 | stard14 | 14:32930072-32937770 | low1C | ctl | OK | 4,89689 | 9,21726 | 0,912473 | 2,83159 | 0,00005 | 0,00421538 | yes |
| ENSDARG00000090893 | HIST1H4C | 7:5787632-5787944 | low1C | ctl | OK | 23,3181 | 43,7821 | 0,908892 | 1,96363 | 0,0008 | 0,041877 | yes |
| ENSDARG00000019702 | aldocb | 21:39585911-39616539 | low1C | ctl | OK | 12,8786 | 24,1272 | 0,90568 | 3,45852 | 0,00005 | 0,00421538 | yes |
| ENSDARG00000076839 | ftr86 | 15:545649-555950 | low1C | ctl | OK | 5,15235 | 9,5572 | 0,891358 | 2,79072 | 0,00005 | 0,00421538 | yes |
| ENSDARG00000076129 | si:ch73-36p18.3 | 25:34619243-34619618 | low1C | ctl | OK | 32,7915 | 60,5953 | 0,885884 | 2,36776 | 0,00005 | 0,00421538 | yes |
| ENSDARG00000100697 | si:ch73-52f24.4 | 16:51328511-51349886 | low1C | ctl | OK | 36,5084 | 67,3748 | 0,883981 | 3,17678 | 0,00005 | 0,00421538 | yes |
| ENSDARG00000053087 | mthfr | 8:47228640-47262086 | low1C | ctl | OK | 3,9068 | 7,1398 | 0,869896 | 2,53705 | 0,00005 | 0,00421538 | yes |
| ENSDARG00000035852 | CU469544.1 | 7:52691499-52692399 | low1C | ctl | OK | 8,14674 | 14,8332 | 0,864536 | 2,13 | 0,0005 | 0,0292267 | yes |
| ENSDARG00000093494 | si:ch211-217k17.9 | 1:51757706-51765926 | low1C | ctl | OK | 14,3483 | 25,9265 | 0,853545 | 2,66141 | 0,00005 | 0,00421538 | yes |
| ENSDARG00000070617 | vhl | 6:40442385-40457573 | low1C | ctl | OK | 4,58671 | 8,21388 | 0,840603 | 2,00688 | 0,00075 | 0,0399757 | yes |
| ENSDARG00000051770 | si:dkey-108k21.19 | 25:34542495-34542882 | low1C | ctl | OK | 19,4709 | 34,8262 | 0,838859 | 1,98769 | 0,00035 | 0,021842 | yes |
| ENSDARG00000037618 | ddit4 | 12:48356810-48358937 | low1C | ctl | OK | 55,125 | 98,2744 | 0,83411 | 3,01043 | 0,00005 | 0,00421538 | yes |

| | | | | | | | | | | | | |
|---|---|---|---|---|---|---|---|---|---|---|---|---|
| ENSDARG00000036965 | rnf24 | 1:41066428-41116690 | low1C | ctl | OK | 3,30593 | 5,8818 | 0,831202 | 2,46664 | 0,00005 | 0,00421538 | yes |
| ENSDARG00000013335 | anxa6 | 14:50263573-50372929 | low1C | ctl | OK | 16,8196 | 29,6845 | 0,819567 | 2,59902 | 0,00005 | 0,00421538 | yes |
| ENSDARG00000103147 | wu:fe11b02 | 7:6227972-6228347 | low1C | ctl | OK | 29,6182 | 51,5553 | 0,799636 | 2,09511 | 0,00035 | 0,021842 | yes |
| ENSDARG00000060962 | btr04 | 7:15924704-15943187 | low1C | ctl | OK | 5,3829 | 9,28738 | 0,786888 | 1,94978 | 0,00085 | 0,0434566 | yes |
| ENSDARG00000103162 | CABZ01046954.1 | 25:32784668-32790239 | low1C | ctl | OK | 3,80662 | 6,49972 | 0,771866 | 1,95741 | 0,00075 | 0,0399757 | yes |
| ENSDARG00000074667 | akt1s1 | 3:30059576-30069715 | low1C | ctl | OK | 40,3324 | 68,5166 | 0,764515 | 3,22962 | 0,00005 | 0,00421538 | yes |
| ENSDARG00000012499 | per1b | 7:50547394-50571221 | low1C | ctl | OK | 2,26439 | 3,77689 | 0,738078 | 2,53501 | 0,00005 | 0,00421538 | yes |
| ENSDARG00000071345 | mgst2 | 14:47229403-47232807 | low1C | ctl | OK | 19,6339 | 32,3769 | 0,721617 | 2,36787 | 0,0001 | 0,00772511 | yes |
| ENSDARG00000004748 | zgc:100868 | 3:39331321-39346662 | low1C | ctl | OK | 8,64695 | 14,2052 | 0,716159 | 2,66711 | 0,00005 | 0,00421538 | yes |
| ENSDARG00000089039 | zgc:173585 | 25:34630717-34631309 | low1C | ctl | OK | 38,0346 | 62,2254 | 0,710193 | 2,38365 | 0,00005 | 0,00421538 | yes |
| ENSDARG00000102806 | zgc:171759 | 7:5839127-5839856 | low1C | ctl | OK | 12,0105 | 19,3469 | 0,687805 | 1,90164 | 0,00095 | 0,0464045 | yes |
| ENSDARG00000051778 | zgc:194989 | 25:34601338-34601825 | low1C | ctl | OK | 30,4689 | 48,5331 | 0,671629 | 1,98471 | 0,00035 | 0,021842 | yes |
| ENSDARG00000077587 | si:dkey-108k21.24 | 25:34645564-34645975 | low1C | ctl | OK | 43,0435 | 68,4009 | 0,668219 | 2,01255 | 0,0007 | 0,0382405 | yes |
| ENSDARG00000034203 | si:dkey-108k21.9 | 25:34543986-34544363 | low1C | ctl | OK | 63,5599 | 100,52 | 0,661299 | 2,06462 | 0,0004 | 0,0243556 | yes |
| ENSDARG00000100783 | PRTG (2 of 2) | KN150056.1:4017-28432 | low1C | ctl | OK | 7,65722 | 12,0677 | 0,656261 | 2,11916 | 0,00045 | 0,0270247 | yes |

| | | | | | | | | | | | | |
|---|---|---|---|---|---|---|---|---|---|---|---|---|
| **ENSDARG00000101251** | **ldha** | **25:25028200-25099874** | **low1C** | **ctl** | **OK** | **22,268** | **34,963** | **0,650857** | **1,97725** | **0,0008** | **0,041877** | **yes** |
| **ENSDARG00000008363** | **mcl1b** | **16:29651325-29656830** | **low1C** | **ctl** | **OK** | **31,9045** | **49,9816** | **0,647636** | **2,20464** | **0,0001** | **0,00772511** | **yes** |
| **ENSDARG00000040284** | **si:dkey-79d12.5** | **16:24904085-24917006** | **low1C** | **ctl** | **OK** | **3,21517** | **5,01694** | **0,641914** | **2,03937** | **0,00035** | **0,021842** | **yes** |
| **ENSDARG00000100185** | **elovl7b** | **KN150589.1:3099-5397** | **low1C** | **ctl** | **OK** | **12,5689** | **19,3368** | **0,62149** | **2,11024** | **0,0004** | **0,0243556** | **yes** |
| **ENSDARG00000094744** | **si:ch211-113a14.15** | **25:35784227-35784648** | **low1C** | **ctl** | **OK** | **52,3859** | **80,5664** | **0,621001** | **1,97256** | **0,0005** | **0,0292267** | **yes** |
| **ENSDARG00000030905** | **cited2** | **20:37012263-37014791** | **low1C** | **ctl** | **OK** | **11,7017** | **17,5106** | **0,581508** | **2,12795** | **0,00025** | **0,0166061** | **yes** |
| **ENSDARG00000010085** | **p4ha2** | **21:43102683-43136621** | **low1C** | **ctl** | **OK** | **19,9216** | **29,4239** | **0,562657** | **2,07897** | **0,00025** | **0,0166061** | **yes** |
| **ENSDARG00000015822** | **sesn3** | **15:35185037-35234766** | **low1C** | **ctl** | **OK** | **8,83073** | **12,9404** | **0,551275** | **2,11481** | **0,0002** | **0,0139175** | **yes** |
| **ENSDARG00000022456** | **eno1a** | **23:22729925-22751447** | **low1C** | **ctl** | **OK** | **58,8905** | **85,2504** | **0,533671** | **1,91561** | **0,00055** | **0,0317263** | **yes** |
| **ENSDARG00000011665** | **aldoaa** | **3:39425140-39435194** | **low1C** | **ctl** | **OK** | **345,1** | **490,856** | **0,508285** | **2,12866** | **0,00035** | **0,021842** | **yes** |
| **ENSDARG00000043973** | **znf292b** | **20:207905-223285** | **low1C** | **ctl** | **OK** | **7,06148** | **9,85044** | **0,480218** | **1,97757** | **0,00035** | **0,021842** | **yes** |
| **ENSDARG00000102808** | **calr3b** | **2:55660711-55673493** | **low1C** | **ctl** | **OK** | **82,6522** | **61,1771** | **-0,434062** | **-1,8691** | **0,00085** | **0,0434566** | **yes** |
| **ENSDARG00000044914** | **suclg2** | **11:16603543-16840555** | **low1C** | **ctl** | **OK** | **43,2517** | **31,3492** | **-0,464331** | **-1,94779** | **0,00095** | **0,0464045** | **yes** |
| **ENSDARG00000001014** | **myh9b** | **3:5177801-5318329** | **low1C** | **ctl** | **OK** | **73,373** | **53,0529** | **-0,467817** | **-2,01202** | **0,00035** | **0,021842** | **yes** |
| **ENSDARG00000101816** | **col5a3b** | **1:58545606-58581062** | **low1C** | **ctl** | **OK** | **26,7759** | **19,3437** | **-0,469075** | **-1,94513** | **0,00085** | **0,0434566** | **yes** |

| | | | | | | | | | | | | |
|---|---|---|---|---|---|---|---|---|---|---|---|---|
| ENSDARG00000008697 | epas1a | 12:25684398-25738189 | low1C | ctl | OK | 11,6985 | 8,41662 | -0,475013 | -1,95049 | 0,0006 | 0,0340505 | yes |
| ENSDARG00000042780 | apoba | 17:30687104-30711003 | low1C | ctl | OK | 22,0712 | 15,8232 | -0,480121 | -2,05623 | 0,0003 | 0,0193412 | yes |
| ENSDARG00000059227 | fabp1b.1 | 8:933677-940431 | low1C | ctl | OK | 1676,01 | 1195,29 | -0,487671 | -2,09021 | 0,0002 | 0,0139175 | yes |
| ENSDARG00000062054 | cpt1ab | 25:23494441-23546729 | low1C | ctl | OK | 13,3451 | 9,44604 | -0,498528 | -1,93038 | 0,0007 | 0,0382405 | yes |
| ENSDARG00000098584 | ndufa13 | KN149883.1:2234-5251 | low1C | ctl | OK | 406,434 | 286,885 | -0,502549 | -1,93905 | 0,00055 | 0,0317263 | yes |
| ENSDARG00000012002 | slc38a7 | 7:51520670-51539701 | low1C | ctl | OK | 19,261 | 13,5553 | -0,506828 | -1,97617 | 0,0006 | 0,0340505 | yes |
| ENSDARG00000074656 | ctssb.1 | 16:29574509-29578811 | low1C | ctl | OK | 112,247 | 78,8843 | -0,508866 | -2,11685 | 0,0002 | 0,0139175 | yes |
| ENSDARG00000101334 | ctsc | 15:43682885-43704485 | low1C | ctl | OK | 80,0405 | 55,5588 | -0,526714 | -2,25314 | 0,0001 | 0,00772511 | yes |
| ENSDARG00000076838 | apom | 2:42379322-42385143 | low1C | ctl | OK | 85,5814 | 59,3823 | -0,527263 | -2,05363 | 0,00085 | 0,0434566 | yes |
| ENSDARG00000036848 | slc43a2a | 15:28435946-28475836 | low1C | ctl | OK | 28,9225 | 20,0518 | -0,528458 | -2,12587 | 0,00015 | 0,01096 | yes |
| ENSDARG00000008637 | mtp | 1:11419100-11439741 | low1C | ctl | OK | 25,5488 | 17,6996 | -0,52954 | -1,90501 | 0,00095 | 0,0464045 | yes |
| ENSDARG00000090850 | serpina1l | 20:10550784-10553957 | low1C | ctl | OK | 46,0278 | 31,8004 | -0,53346 | -2,14716 | 0,00035 | 0,021842 | yes |
| ENSDARG00000093549 | sepp1a | 8:31426219-31434102 | low1C | ctl | OK | 161,647 | 111,37 | -0,537488 | -2,1831 | 0,00005 | 0,00421538 | yes |
| ENSDARG00000103271 | pla2g15 | 25:13592659-13607426 | low1C | ctl | OK | 27,1849 | 18,6817 | -0,541183 | -2,1619 | 0,0003 | 0,0193412 | yes |
| ENSDARG00000099301 | CDC37 | KN149781.1:4958-8644 | low1C | ctl | OK | 310,37 | 213,253 | -0,541425 | -2,33776 | 0,0002 | 0,0139175 | yes |

| | | | | | | | | | | | | |
|---|---|---|---|---|---|---|---|---|---|---|---|---|
| ENSDARG00000045516 | itih2 | 4:25192373-25226770 | low1C | ctl | OK | 25,4969 | 17,4664 | -0,545736 | -1,98864 | 0,0007 | 0,0382405 | yes |
| ENSDARG00000036893 | f13a1b | 7:8024804-8098285 | low1C | ctl | OK | 15,9014 | 10,839 | -0,55292 | -2,06521 | 0,0003 | 0,0193412 | yes |
| ENSDARG00000061383 | serpinf2b | 15:26608142-26619776 | low1C | ctl | OK | 13,9499 | 9,49966 | -0,554306 | -1,8815 | 0,001 | 0,0481758 | yes |
| ENSDARG00000036074 | cebpa | 7:37813727-37816607 | low1C | ctl | OK | 17,4648 | 11,8888 | -0,554841 | -2,14541 | 0,00035 | 0,021842 | yes |
| ENSDARG00000056640 | rpia | 13:13799270-13813296 | low1C | ctl | OK | 69,7565 | 47,4777 | -0,555078 | -1,87262 | 0,0008 | 0,041877 | yes |
| ENSDARG00000088439 | gm2a | 14:25927109-25930546 | low1C | ctl | OK | 37,2425 | 25,2348 | -0,561534 | -2,05785 | 0,0002 | 0,0139175 | yes |
| ENSDARG00000016745 | slc35f6 | 20:26005108-26011553 | low1C | ctl | OK | 42,4158 | 28,6491 | -0,566113 | -2,33874 | 0,00005 | 0,00421538 | yes |
| ENSDARG00000003570 | hsp90b1 | 4:9835548-9842920 | low1C | ctl | OK | 48,5198 | 32,719 | -0,568446 | -2,37214 | 0,00005 | 0,00421538 | yes |
| ENSDARG00000018529 | lipf | 12:17036767-17079154 | low1C | ctl | OK | 14,9567 | 10,0834 | -0,568813 | -1,96969 | 0,00075 | 0,0399757 | yes |
| ENSDARG00000101239 | hsd17b4 | 8:190534-214498 | low1C | ctl | OK | 8,60588 | 5,78275 | -0,573569 | -1,95663 | 0,0009 | 0,0447093 | yes |
| ENSDARG00000090912 | npc2 | 17:43031044-43041345 | low1C | ctl | OK | 312,112 | 209,711 | -0,573663 | -2,47342 | 0,00005 | 0,00421538 | yes |
| ENSDARG00000098645 | rgn | 6:8895449-8904447 | low1C | ctl | OK | 14,088 | 9,39852 | -0,583962 | -1,91649 | 0,0009 | 0,0447093 | yes |
| ENSDARG00000039459 | qsox1 | 8:14465904-14517080 | low1C | ctl | OK | 8,2131 | 5,46312 | -0,588201 | -1,98653 | 0,0005 | 0,0292267 | yes |
| ENSDARG00000042010 | PKLR | 16:54629440-54634651 | low1C | ctl | OK | 18,2168 | 12,1114 | -0,588904 | -2,04895 | 0,00045 | 0,0270247 | yes |
| ENSDARG00000037307 | gnpda1 | 14:23411966-23416458 | low1C | ctl | OK | 24,9583 | 16,5034 | -0,596758 | -2,01853 | 0,00055 | 0,0317263 | yes |

| | | | | | | | | | | | | |
|---|---|---|---|---|---|---|---|---|---|---|---|---|
| **ENSDARG00000032976** | **cmlc1** | **5:63486420-63487216** | **low1C** | **ctl** | **OK** | **57,5792** | **37,9796** | **-0,600323** | **-2,20435** | **0,0002** | **0,0139175** | **yes** |
| **ENSDARG00000012729** | **hcls1** | **4:22579351-22590686** | **low1C** | **ctl** | **OK** | **5,77728** | **3,80711** | **-0,601696** | **-1,83482** | **0,001** | **0,0481758** | **yes** |
| **ENSDARG00000105142** | **tcirg1b** | **14:16471654-16504836** | **low1C** | **ctl** | **OK** | **14,1276** | **9,29065** | **-0,60467** | **-2,29354** | **0,00005** | **0,00421538** | **yes** |
| **ENSDARG00000023082** | **krt1-19d** | **19:5408166-5416298** | **low1C** | **ctl** | **OK** | **26,3392** | **17,3202** | **-0,604763** | **-2,15658** | **0,0004** | **0,0243556** | **yes** |
| **ENSDARG00000003931** | **cndp2** | **19:21216736-21238776** | **low1C** | **ctl** | **OK** | **12,6578** | **8,32284** | **-0,604883** | **-2,08788** | **0,0003** | **0,0193412** | **yes** |
| **ENSDARG00000075016** | **apobb.2** | **20:53634247-53664835** | **low1C** | **ctl** | **OK** | **8,37931** | **5,5062** | **-0,605774** | **-2,43677** | **0,00005** | **0,00421538** | **yes** |
| **ENSDARG00000100589** | **ZNF830** | **KN150544.1:0-282** | **low1C** | **ctl** | **OK** | **227,396** | **149,338** | **-0,606623** | **-2,00609** | **0,00025** | **0,0166061** | **yes** |
| **ENSDARG00000103347** | **cyp2aa3** | **23:42516640-42528380** | **low1C** | **ctl** | **OK** | **23,4633** | **15,3815** | **-0,60921** | **-2,23865** | **0,00015** | **0,01096** | **yes** |
| **ENSDARG00000063518** | **CPN2** | **2:50629-53978** | **low1C** | **ctl** | **OK** | **15,1543** | **9,91989** | **-0,611336** | **-2,21577** | **0,00015** | **0,01096** | **yes** |
| **ENSDARG00000090286** | **serpina1** | **20:10556859-10560171** | **low1C** | **ctl** | **OK** | **89,4232** | **57,8653** | **-0,62795** | **-2,45919** | **0,00005** | **0,00421538** | **yes** |
| **ENSDARG00000099389** | **cdo1** | **KN149707.1:3860-5958** | **low1C** | **ctl** | **OK** | **152,655** | **98,7188** | **-0,628881** | **-2,47779** | **0,00005** | **0,00421538** | **yes** |
| **ENSDARG00000053973** | **fetub** | **22:37934446-37944777** | **low1C** | **ctl** | **OK** | **970,509** | **627,092** | **-0,630064** | **-2,49604** | **0,00005** | **0,00421538** | **yes** |
| **ENSDARG00000099200** | **zgc:123103** | **19:42978261-42981901** | **low1C** | **ctl** | **OK** | **59,2257** | **37,9883** | **-0,640666** | **-2,52319** | **0,00005** | **0,00421538** | **yes** |
| **ENSDARG00000042684** | **serpinc1** | **22:15995940-16001921** | **low1C** | **ctl** | **OK** | **33,3716** | **21,2769** | **-0,64933** | **-2,63956** | **0,00005** | **0,00421538** | **yes** |
| **ENSDARG00000093702** | **si:dkey-126g1.9** | **23:32007051-32014381** | **low1C** | **ctl** | **OK** | **88,9135** | **56,5944** | **-0,651742** | **-2,68291** | **0,00005** | **0,00421538** | **yes** |

| | | | | | | | | | | | | |
|---|---|---|---|---|---|---|---|---|---|---|---|---|
| ENSDARG00000015866 | apoa2 | 16:24063945-24065322 | low1C | ctl | OK | 1613,07 | 1022,26 | -0,658048 | -2,76871 | 0,00005 | 0,00421538 | yes |
| ENSDARG00000079501 | si:ch211-149l1.2 | 1:49031535-49054672 | low1C | ctl | OK | 5,92909 | 3,72392 | -0,670989 | -2,00922 | 0,0005 | 0,0292267 | yes |
| ENSDARG00000094426 | her4.2 | 23:21528600-21534589 | low1C | ctl | OK | 12,4091 | 7,79203 | -0,671324 | -1,94793 | 0,0005 | 0,0292267 | yes |
| ENSDARG00000069583 | CNDP1 | 16:55296447-55320715 | low1C | ctl | OK | 99,3441 | 61,552 | -0,690627 | -2,41782 | 0,00005 | 0,00421538 | yes |
| ENSDARG00000044125 | txn | 7:24086073-24093669 | low1C | ctl | OK | 164,179 | 101,674 | -0,691309 | -2,76479 | 0,00005 | 0,00421538 | yes |
| ENSDARG00000039605 | mat1a | 13:18497927-18506252 | low1C | ctl | OK | 68,2073 | 42,1458 | -0,694539 | -2,96607 | 0,00005 | 0,00421538 | yes |
| ENSDARG00000044685 | nr0b2a | 16:55167504-55172525 | low1C | ctl | OK | 25,5447 | 15,7168 | -0,700714 | -2,82 | 0,00005 | 0,00421538 | yes |
| ENSDARG00000102241 | zgc:136410 | 21:44510840-44518121 | low1C | ctl | OK | 244,221 | 150,241 | -0,700912 | -2,99312 | 0,00005 | 0,00421538 | yes |
| ENSDARG00000043843 | akap7 | 20:2402636-2462287 | low1C | ctl | OK | 47,721 | 29,3512 | -0,701205 | -2,18111 | 0,00025 | 0,0166061 | yes |
| ENSDARG00000102207 | CABZ01102578.1 | KN149735.1:21-4011 | low1C | ctl | OK | 49,3629 | 30,3568 | -0,701407 | -2,56606 | 0,00005 | 0,00421538 | yes |
| ENSDARG00000102356 | scp2b | 23:41935798-41959885 | low1C | ctl | OK | 15,2217 | 9,35947 | -0,701635 | -2,05842 | 0,00075 | 0,0399757 | yes |
| ENSDARG00000056151 | tyrp1b | 1:18639899-18653111 | low1C | ctl | OK | 12,4583 | 7,62223 | -0,708821 | -2,17012 | 0,0003 | 0,0193412 | yes |
| ENSDARG00000020143 | pah | 4:17402703-17420557 | low1C | ctl | OK | 9,93856 | 6,07977 | -0,70902 | -2,28832 | 0,0001 | 0,00772511 | yes |
| ENSDARG00000007421 | ftcd | 22:12746099-12761755 | low1C | ctl | OK | 11,4088 | 6,85559 | -0,734794 | -2,31605 | 0,00005 | 0,00421538 | yes |
| ENSDARG00000040295 | apoeb | 16:24045711-24048429 | low1C | ctl | OK | 2292,66 | 1373,62 | -0,739041 | -2,58954 | 0,00005 | 0,00421538 | yes |

| | | | | | | | | | | | | |
|---|---|---|---|---|---|---|---|---|---|---|---|---|
| ENSDARG00000 019713 | oatx | 21:44088982-44109518 | low1C | ctl | OK | 6,18077 | 3,69922 | -0,740565 | - 2,29352 | 0,0002 | 0,01391 75 | yes |
| ENSDARG00000 043154 | ucp2 | 10:36706162-36738764 | low1C | ctl | OK | 159,602 | 95,216 | -0,745204 | - 3,11804 | 0,00005 | 0,00421 538 | yes |
| ENSDARG00000 026109 | slc48a1b | 6:38898621-38904962 | low1C | ctl | OK | 9,87869 | 5,86775 | -0,751513 | - 1,99431 | 0,00075 | 0,03997 57 | yes |
| ENSDARG00000 009018 | rhbg | 16:29331172-29342541 | low1C | ctl | OK | 7,91003 | 4,68476 | -0,755707 | -2,6598 | 0,00005 | 0,00421 538 | yes |
| ENSDARG00000 008931 | renbp | 8:36998539-37011466 | low1C | ctl | OK | 12,3043 | 7,25395 | -0,762329 | - 2,53027 | 0,00005 | 0,00421 538 | yes |
| ENSDARG00000 004296 | ambp | 10:10843325-10865634 | low1C | ctl | OK | 59,7223 | 35,0215 | -0,770027 | - 2,22904 | 0,0001 | 0,00772 511 | yes |
| ENSDARG00000 098586 | zgc:194887 | 17:443532-449054 | low1C | ctl | OK | 12,3761 | 7,2385 | -0,773793 | - 2,02017 | 0,00095 | 0,04640 45 | yes |
| ENSDARG00000 021004 | c5 | 5:62764793-62816203 | low1C | ctl | OK | 7,10854 | 4,13541 | -0,781524 | - 2,84672 | 0,00005 | 0,00421 538 | yes |
| ENSDARG00000 038668 | gbp1 | 2:42171489-42185905 | low1C | ctl | OK | 4,46901 | 2,59503 | -0,784203 | - 2,04376 | 0,0004 | 0,02435 56 | yes |
| ENSDARG00000 011701 | ctsll | 5:23802076-23805867 | low1C | ctl | OK | 98,5437 | 57,0847 | -0,787659 | - 3,22561 | 0,00005 | 0,00421 538 | yes |
| ENSDARG00000 058102 | sardh | 10:10429089-10603580 | low1C | ctl | OK | 15,7799 | 9,09355 | -0,79517 | - 3,02206 | 0,00005 | 0,00421 538 | yes |
| ENSDARG00000 007988 | masp2 | 23:28843683-28869616 | low1C | ctl | OK | 3,20656 | 1,83667 | -0,803938 | - 1,91301 | 0,0009 | 0,04470 93 | yes |
| ENSDARG00000 032862 | ugt5g1 | 7:22420803-22425829 | low1C | ctl | OK | 2,92023 | 1,67056 | -0,805751 | - 1,96419 | 0,00085 | 0,04345 66 | yes |
| ENSDARG00000 100917 | slc30a8 | 19:46751129-46769977 | low1C | ctl | OK | 3,53564 | 2,01954 | -0,807942 | - 2,35603 | 0,00005 | 0,00421 538 | yes |
| ENSDARG00000 104207 | pcyt1bb | 21:40595567-40607603 | low1C | ctl | OK | 4,38927 | 2,45944 | -0,83565 | - 2,02171 | 0,0007 | 0,03824 05 | yes |

| | | | | | | | | | | | | |
|---|---|---|---|---|---|---|---|---|---|---|---|---|
| ENSDARG00000041595 | ces3 | 18:17429187-17437240 | low1C | ctl | OK | 4,79724 | 2,68507 | -0,837244 | -2,0696 | 0,00025 | 0,0166061 | yes |
| ENSDARG00000036041 | f2 | 7:38479612-38488900 | low1C | ctl | OK | 22,7069 | 12,6385 | -0,845303 | -2,56315 | 0,00005 | 0,00421538 | yes |
| ENSDARG00000019986 | grhprb | 1:19142865-19152602 | low1C | ctl | OK | 25,1699 | 13,9388 | -0,852597 | -2,79985 | 0,00005 | 0,00421538 | yes |
| ENSDARG00000018264 | trim101 | 23:17528347-17543803 | low1C | ctl | OK | 4,61844 | 2,54509 | -0,859691 | -2,10414 | 0,00025 | 0,0166061 | yes |
| ENSDARG00000039516 | c8a | 2:2374645-2401596 | low1C | ctl | OK | 7,39509 | 4,05234 | -0,867813 | -1,93952 | 0,00085 | 0,0434566 | yes |
| ENSDARG00000015662 | pla2g12b | 13:4543111-4555009 | low1C | ctl | OK | 220,906 | 120,727 | -0,871686 | -2,82078 | 0,00005 | 0,00421538 | yes |
| ENSDARG00000017261 | gdpd1 | 15:16950173-16958853 | low1C | ctl | OK | 21,78 | 11,9011 | -0,871915 | -2,31284 | 0,00005 | 0,00421538 | yes |
| ENSDARG00000039099 | zgc:113413 | 7:8127066-8135474 | low1C | ctl | OK | 11,5288 | 6,28308 | -0,875697 | -2,39331 | 0,0001 | 0,00772511 | yes |
| ENSDARG00000102456 | cfhl4 | 22:23587102-23598808 | low1C | ctl | OK | 27,0964 | 14,7468 | -0,877695 | -3,06366 | 0,00005 | 0,00421538 | yes |
| ENSDARG00000074362 | FUT9 (10 of 14) | 3:51837689-51858158 | low1C | ctl | OK | 1,36298 | 0,741137 | -0,87895 | -2,00294 | 0,00055 | 0,0317263 | yes |
| ENSDARG00000021208 | serpind1 | 8:1736412-1833109 | low1C | ctl | OK | 2,63123 | 1,42265 | -0,887152 | -2,03363 | 0,0007 | 0,0382405 | yes |
| ENSDARG00000043180 | gpd1b | 22:6966199-6977128 | low1C | ctl | OK | 38,5165 | 20,8083 | -0,888317 | -3,35176 | 0,00005 | 0,00421538 | yes |
| ENSDARG00000042379 | TMEM120A (2 of 2) | 15:16937667-16948890 | low1C | ctl | OK | 3,25067 | 1,74493 | -0,897569 | -1,96797 | 0,001 | 0,0481758 | yes |
| ENSDARG00000091465 | si:dkey-66a8.7 | 6:37463894-37482284 | low1C | ctl | OK | 8,37453 | 4,4659 | -0,907057 | -2,06011 | 0,00075 | 0,0399757 | yes |
| ENSDARG00000101160 | apoa4a | 19:10927027-10931909 | low1C | ctl | OK | 57,2388 | 30,5163 | -0,907416 | -3,35444 | 0,00005 | 0,00421538 | yes |

| | | | | | | | | | | | | |
|---|---|---|---|---|---|---|---|---|---|---|---|---|
| ENSDARG00000012640 | cideb | 7:23744357-23751183 | low1C | ctl | OK | 10,2017 | 5,4256 | -0,910952 | -2,42492 | 0,00005 | 0,00421538 | yes |
| ENSDARG00000053831 | vtnb | 21:39022453-39036049 | low1C | ctl | OK | 15,824 | 8,3232 | -0,926906 | -3,11322 | 0,00005 | 0,00421538 | yes |
| ENSDARG00000012694 | c3a.1 | 1:55460925-55507680 | low1C | ctl | OK | 38,0099 | 19,9805 | -0,927787 | -3,88562 | 0,00005 | 0,00421538 | yes |
| ENSDARG00000028396 | fkbp5 | 6:41098521-41118517 | low1C | ctl | OK | 2,00912 | 1,05375 | -0,931027 | -1,9438 | 0,0009 | 0,0447093 | yes |
| ENSDARG00000027529 | hmox1a | 3:25880610-25886721 | low1C | ctl | OK | 31,1266 | 16,2143 | -0,940885 | -3,43317 | 0,00005 | 0,00421538 | yes |
| ENSDARG00000055278 | cfb | 21:27379588-27394702 | low1C | ctl | OK | 11,3318 | 5,83059 | -0,958665 | -3,16588 | 0,00005 | 0,00421538 | yes |
| ENSDARG00000069828 | ABHD12B (1 of 2) | 13:9546520-9554556 | low1C | ctl | OK | 5,8994 | 3,00715 | -0,972168 | -2,25808 | 0,00015 | 0,01096 | yes |
| ENSDARG00000055504 | CD68 | 7:19231023-19239661 | low1C | ctl | OK | 42,9579 | 21,8613 | -0,974547 | -3,80529 | 0,00005 | 0,00421538 | yes |
| ENSDARG00000021250 | slc25a48 | 14:25293357-25301757 | low1C | ctl | OK | 7,05456 | 3,56837 | -0,98329 | -2,48427 | 0,00005 | 0,00421538 | yes |
| ENSDARG00000045299 | vmo1b | 21:1592356-1600152 | low1C | ctl | OK | 8,92733 | 4,43559 | -1,0091 | -2,63154 | 0,00005 | 0,00421538 | yes |
| ENSDARG00000036966 | hsd3b7 | 3:32612231-32646536 | low1C | ctl | OK | 4,56532 | 2,25638 | -1,0167 | -2,48412 | 0,0001 | 0,00772511 | yes |
| ENSDARG00000016793 | crybb1l2 | 1:43720616-43745614 | low1C | ctl | OK | 11,1663 | 5,50707 | -1,0198 | -2,54998 | 0,0001 | 0,00772511 | yes |
| ENSDARG00000102594 | zgc:194627 | 22:5048518-5057287 | low1C | ctl | OK | 5,7333 | 2,82455 | -1,02135 | -2,17409 | 0,00025 | 0,0166061 | yes |
| ENSDARG00000023111 | plg | 20:42791377-42805774 | low1C | ctl | OK | 16,3043 | 8,00017 | -1,02715 | -3,80501 | 0,00005 | 0,00421538 | yes |
| ENSDARG00000086998 | NNMT | 11:11991086-11996233 | low1C | ctl | OK | 3,21547 | 1,57418 | -1,03043 | -2,53945 | 0,00005 | 0,00421538 | yes |

| | | | | | | | | | | | | |
|---|---|---|---|---|---|---|---|---|---|---|---|---|
| ENSDARG00000070038 | rbp2a | 15:5815606-5827155 | low1C | ctl | OK | 25,941 | 12,6644 | -1,03445 | -2,82306 | 0,00005 | 0,00421538 | yes |
| ENSDARG00000068883 | tspan13a | 15:34734245-34741801 | low1C | ctl | OK | 1,90561 | 0,92527 | -1,0423 | -2,53098 | 0,00005 | 0,00421538 | yes |
| ENSDARG00000040298 | apoa4b.1 | 16:23999037-24033538 | low1C | ctl | OK | 459,546 | 222,625 | -1,04559 | -3,5463 | 0,00005 | 0,00421538 | yes |
| ENSDARG00000090637 | myh6 | 20:53772084-53778019 | low1C | ctl | OK | 1,1424 | 0,547594 | -1,06089 | -2,51168 | 0,00005 | 0,00421538 | yes |
| ENSDARG00000036171 | rnasel3 | 14:38313985-38316149 | low1C | ctl | OK | 8,43185 | 4,01492 | -1,07048 | -2,57261 | 0,00005 | 0,00421538 | yes |
| ENSDARG00000103875 | CABZ01048402.2 | KN149946.1:4414-12640 | low1C | ctl | OK | 10,4874 | 4,98327 | -1,07349 | -3,17466 | 0,00005 | 0,00421538 | yes |
| ENSDARG00000100442 | cfh | 22:23615378-23721721 | low1C | ctl | OK | 15,0285 | 7,12577 | -1,07659 | -3,42888 | 0,00005 | 0,00421538 | yes |
| ENSDARG00000006220 | ugt1ab | 9:9998613-10032338 | low1C | ctl | OK | 2,541 | 1,176 | -1,11151 | -2,07416 | 0,0002 | 0,0139175 | yes |
| ENSDARG00000090722 | C20H6orf58 (1 of 2) | 20:1391366-1400179 | low1C | ctl | OK | 9,92906 | 4,57242 | -1,1187 | -2,99203 | 0,00005 | 0,00421538 | yes |
| ENSDARG00000019260 | dhrs9 | 9:49008552-49038903 | low1C | ctl | OK | 4,13832 | 1,90015 | -1,12293 | -2,37763 | 0,00015 | 0,01096 | yes |
| ENSDARG00000035257 | akr1a1a | 5:40920885-40931476 | low1C | ctl | OK | 6,89005 | 3,16205 | -1,12366 | -2,56243 | 0,00005 | 0,00421538 | yes |
| ENSDARG00000020741 | fga | 1:9379389-9387428 | low1C | ctl | OK | 61,1514 | 28,0576 | -1,124 | -3,75197 | 0,00005 | 0,00421538 | yes |
| ENSDARG00000103800 | prkar1b | 3:12853207-12927273 | low1C | ctl | OK | 2,67944 | 1,22466 | -1,12955 | -2,09845 | 0,00075 | 0,0399757 | yes |
| ENSDARG00000070394 | uroc1 | 6:40041997-40061183 | low1C | ctl | OK | 2,36611 | 1,06975 | -1,14524 | -2,56455 | 0,00005 | 0,00421538 | yes |
| ENSDARG00000070168 | sod3a | 7:71096759-71105633 | low1C | ctl | OK | 12,8364 | 5,76171 | -1,15567 | -3,17641 | 0,00005 | 0,00421538 | yes |

| | | | | | | | | | | | | |
|---|---|---|---|---|---|---|---|---|---|---|---|---|
| ENSDARG00000097606 | tmem82 | 23:24558931-24562155 | low1C | ctl | OK | 2,37724 | 1,0541 | -1,17327 | -1,96684 | 0,0009 | 0,0447093 | yes |
| ENSDARG00000030508 | CCDC134 (1 of 2) | 3:662600-671629 | low1C | ctl | OK | 13,1682 | 5,83316 | -1,17471 | -3,05492 | 0,00005 | 0,00421538 | yes |
| ENSDARG00000078918 | comtd1 | 13:17541534-17554353 | low1C | ctl | OK | 10,0345 | 4,39039 | -1,19255 | -1,98241 | 0,00095 | 0,0464045 | yes |
| ENSDARG00000070918 | FETUB (2 of 2) | 22:38276173-38287021 | low1C | ctl | OK | 4,13355 | 1,80788 | -1,19308 | -2,36985 | 0,00005 | 0,00421538 | yes |
| ENSDARG00000001975 | hsd11b2 | 7:34840574-34854897 | low1C | ctl | OK | 1,54465 | 0,657752 | -1,23167 | -2,38257 | 0,0001 | 0,00772511 | yes |
| ENSDARG00000104576 | PPP1R3G | 24:2827465-2830305 | low1C | ctl | OK | 4,53467 | 1,92605 | -1,23535 | -3,34052 | 0,00005 | 0,00421538 | yes |
| ENSDARG00000062483 | lyve1b | 18:16755246-16758322 | low1C | ctl | OK | 3,18741 | 1,33223 | -1,25854 | -2,06028 | 0,0008 | 0,041877 | yes |
| ENSDARG00000002764 | miox | 18:6773232-6783325 | low1C | ctl | OK | 5,72136 | 2,38499 | -1,26237 | -2,89301 | 0,00005 | 0,00421538 | yes |
| ENSDARG00000078068 | abcg4b | 15:23552464-23572949 | low1C | ctl | OK | 1,12579 | 0,466813 | -1,27002 | -1,7488 | 0,0009 | 0,0447093 | yes |
| ENSDARG00000034063 | CABZ01068208.1 | 17:53294819-53301747 | low1C | ctl | OK | 8,26714 | 3,37669 | -1,29178 | -3,61965 | 0,00005 | 0,00421538 | yes |
| ENSDARG00000061303 | oca2 | 6:37881001-38060286 | low1C | ctl | OK | 0,595362 | 0,240487 | -1,30781 | -2,03503 | 0,0003 | 0,0193412 | yes |
| ENSDARG00000058005 | hgd | 24:23814312-23825872 | low1C | ctl | OK | 3,1484 | 1,26771 | -1,31239 | -2,61482 | 0,00005 | 0,00421538 | yes |
| ENSDARG00000068114 | f11r.2 | 5:29220112-29225693 | low1C | ctl | OK | 3,18278 | 1,26905 | -1,32653 | -2,55111 | 0,00005 | 0,00421538 | yes |
| ENSDARG00000104702 | cat | 25:7318705-7328383 | low1C | ctl | OK | 8,61644 | 3,36905 | -1,35475 | -3,86358 | 0,00005 | 0,00421538 | yes |
| ENSDARG00000043719 | c3a.6 | 1:55360637-55410849 | low1C | ctl | OK | 2,29302 | 0,896012 | -1,35566 | -2,84294 | 0,00005 | 0,00421538 | yes |

| | | | | | | | | | | | | |
|---|---|---|---|---|---|---|---|---|---|---|---|---|
| ENSDARG00000055036 | itih3a | 11:3594938-3609829 | low1C | ctl | OK | 6,79873 | 2,60843 | -1,38208 | -1,98978 | 0,00085 | 0,0434566 | yes |
| ENSDARG00000080001 | si:dkey-208m12.2 | 22:27489647-27499674 | low1C | ctl | OK | 0,63409 | 0,239511 | -1,4046 | -2,4085 | 0,00015 | 0,01096 | yes |
| ENSDARG00000019772 | si:ch1073-280e3.1 | 15:517687-535747 | low1C | ctl | OK | 1,14719 | 0,429375 | -1,41779 | -2,28102 | 0,00015 | 0,01096 | yes |
| ENSDARG00000008969 | fgb | 1:9367651-9375385 | low1C | ctl | OK | 54,0108 | 20,1926 | -1,41942 | -4,86742 | 0,00005 | 0,00421538 | yes |
| ENSDARG00000019532 | fads2 | 25:4007595-4023060 | low1C | ctl | OK | 9,36146 | 3,37539 | -1,47168 | -3,71563 | 0,00005 | 0,00421538 | yes |
| ENSDARG00000069524 | si:dkey-30c15.13 | 18:7436733-7441140 | low1C | ctl | OK | 2,86276 | 1,02679 | -1,47927 | -2,1668 | 0,00065 | 0,0365333 | yes |
| ENSDARG00000095556 | si:dkey-238c7.12 | 18:7607071-7609022 | low1C | ctl | OK | 317,685 | 112,742 | -1,49458 | -5,82955 | 0,00005 | 0,00421538 | yes |
| ENSDARG00000093774 | rbp2b | 2:39050426-39052199 | low1C | ctl | OK | 7,35546 | 2,60887 | -1,49539 | -3,29748 | 0,00005 | 0,00421538 | yes |
| ENSDARG00000037281 | fgg | 1:24978969-24988554 | low1C | ctl | OK | 39,5484 | 13,9832 | -1,49992 | -5,40795 | 0,00005 | 0,00421538 | yes |
| ENSDARG00000038405 | zgc:153921 | 3:1236409-1293074 | low1C | ctl | OK | 3,43906 | 1,20714 | -1,51042 | -2,3636 | 0,00015 | 0,01096 | yes |
| ENSDARG00000041623 | mt2 | 18:17545560-17546913 | low1C | ctl | OK | 148,921 | 52,0557 | -1,51642 | -5,39305 | 0,00005 | 0,00421538 | yes |
| ENSDARG00000058053 | serping1 | 8:13922949-14014644 | low1C | ctl | OK | 5,97046 | 1,99858 | -1,57887 | -2,18677 | 0,0008 | 0,041877 | yes |
| ENSDARG00000098642 | PRKAR1B | 3:12966603-12987150 | low1C | ctl | OK | 4,62288 | 1,53756 | -1,58815 | -2,16312 | 0,0005 | 0,0292267 | yes |
| ENSDARG00000055705 | f5 | 9:34317820-34338811 | low1C | ctl | OK | 0,847765 | 0,279409 | -1,60129 | -3,23789 | 0,00005 | 0,00421538 | yes |
| ENSDARG00000051912 | HPX (2 of 2) | 16:2883517-2903388 | low1C | ctl | OK | 5,81375 | 1,85502 | -1,64803 | -3,59037 | 0,00005 | 0,00421538 | yes |

| | | | | | | | | | | | | |
|---|---|---|---|---|---|---|---|---|---|---|---|---|
| ENSDARG00000053448 | si:ch211-251f6.6 | 10:8086654-8121037 | low1C | ctl | OK | 8,40464 | 2,67507 | -1,65161 | -2,90939 | 0,00005 | 0,00421538 | yes |
| ENSDARG00000059651 | cidec | 6:19142441-19151867 | low1C | ctl | OK | 1,35284 | 0,4201 | -1,68719 | -2,38887 | 0,00025 | 0,0166061 | yes |
| ENSDARG00000104572 | si:dkey-35h6.1 | 14:12730545-12742341 | low1C | ctl | OK | 0,826825 | 0,254983 | -1,69718 | -2,89777 | 0,0001 | 0,00772511 | yes |
| ENSDARG00000023151 | ucp1 | 1:52687139-52869865 | low1C | ctl | OK | 32,2487 | 9,70759 | -1,73206 | -2,19391 | 0,0009 | 0,0447093 | yes |
| ENSDARG00000102722 | itih3b | 11:36910727-36932766 | low1C | ctl | OK | 1,95999 | 0,589179 | -1,73407 | -3,02909 | 0,00005 | 0,00421538 | yes |
| ENSDARG00000095751 | C20H6orf58 (2 of 2) | 20:1377737-1385482 | low1C | ctl | OK | 5,00379 | 1,43982 | -1,79714 | -3,57317 | 0,00005 | 0,00421538 | yes |
| ENSDARG00000104329 | NOTCH4 | 8:19482208-19488256 | low1C | ctl | OK | 2,09065 | 0,583178 | -1,84194 | -2,45134 | 0,0001 | 0,00772511 | yes |
| ENSDARG00000033227 | lect2l | 14:36545209-36546752 | low1C | ctl | OK | 4,88051 | 1,32495 | -1,88109 | -2,92566 | 0,00005 | 0,00421538 | yes |
| ENSDARG00000057498 | habp2 | 12:31598336-31605164 | low1C | ctl | OK | 1,25656 | 0,339267 | -1,88899 | -2,68412 | 0,0001 | 0,00772511 | yes |
| ENSDARG00000004199 | spam1 | 25:27092796-27121034 | low1C | ctl | OK | 2,0474 | 0,516569 | -1,98676 | -3,07535 | 0,00005 | 0,00421538 | yes |
| ENSDARG00000069630 | tat | 18:13194009-13210998 | low1C | ctl | OK | 1,3746 | 0,341749 | -2,008 | -2,954 | 0,00005 | 0,00421538 | yes |
| ENSDARG00000029230 | pnp4b | 16:36854039-36880749 | low1C | ctl | OK | 6,00324 | 1,46967 | -2,03025 | -2,93947 | 0,00005 | 0,00421538 | yes |
| ENSDARG00000005616 | bfb | 21:27401171-27407300 | low1C | ctl | OK | 1,64486 | 0,399621 | -2,04126 | -2,20636 | 0,0006 | 0,0340505 | yes |
| ENSDARG00000079589 | si:dkeyp-73d8.6 | 2:32638458-32641359 | low1C | ctl | OK | 3,96679 | 0,961289 | -2,04493 | -3,01373 | 0,00005 | 0,00421538 | yes |
| ENSDARG00000070892 | si:ch211-262h13.5 | 22:38825803-38832858 | low1C | ctl | OK | 1,83778 | 0,444732 | -2,04696 | -1,99969 | 0,0007 | 0,0382405 | yes |

| | | | | | | | | | | | | |
|---|---|---|---|---|---|---|---|---|---|---|---|---|
| ENSDARG00000057844 | zgc:171470 | 1:56783698-56787039 | low1C | ctl | OK | 0,724686 | 0,174454 | -2,05451 | -2,30173 | 0,0008 | 0,041877 | yes |
| ENSDARG00000036776 | aldh8a1 | 23:31826072-31837222 | low1C | ctl | OK | 3,82032 | 0,909964 | -2,06981 | -3,99034 | 0,00005 | 0,00421538 | yes |
| ENSDARG00000069293 | ahsg2 | 2:10336524-10343921 | low1C | ctl | OK | 4,26364 | 0,988052 | -2,10943 | -3,73366 | 0,00005 | 0,00421538 | yes |
| ENSDARG00000070960 | si:ch211-288g17.4 | 19:9425245-9445417 | low1C | ctl | OK | 2,13304 | 0,482005 | -2,14579 | -1,52113 | 0,0005 | 0,0292267 | yes |
| ENSDARG00000017444 | hal | 25:7830047-7850402 | low1C | ctl | OK | 0,542073 | 0,121139 | -2,16182 | -2,28282 | 0,00075 | 0,0399757 | yes |
| ENSDARG00000075614 | zgc:193682 | 16:24060897-24062096 | low1C | ctl | OK | 2,90979 | 0,649827 | -2,16279 | -2,1112 | 0,00045 | 0,0270247 | yes |
| ENSDARG00000076043 | si:dkeyp-73d8.9 | 2:32659119-32660520 | low1C | ctl | OK | 3,67177 | 0,721929 | -2,34655 | -2,6387 | 0,00005 | 0,00421538 | yes |
| ENSDARG00000003523 | itln3 | 14:16039349-16046556 | low1C | ctl | OK | 2,12752 | 0,418031 | -2,34749 | -2,38528 | 0,00015 | 0,01096 | yes |
| ENSDARG00000026764 | ahsg1 | 22:37920795-37927972 | low1C | ctl | OK | 13,3256 | 2,37475 | -2,48835 | -5,18307 | 0,00005 | 0,00421538 | yes |
| ENSDARG00000089310 | gc | 5:45077667-45124680 | low1C | ctl | OK | 1,9247 | 0,337728 | -2,5107 | -3,01755 | 0,00005 | 0,00421538 | yes |
| ENSDARG00000036833 | upp2 | 9:16160545-16171735 | low1C | ctl | OK | 1,18104 | 0,207058 | -2,51195 | -2,82978 | 0,00005 | 0,00421538 | yes |
| ENSDARG00000012609 | hpx | 9:33029367-33046387 | low1C | ctl | OK | 11,8579 | 2,00551 | -2,56381 | -4,82225 | 0,00005 | 0,00421538 | yes |
| ENSDARG00000033662 | scd | 12:48407392-48421350 | low1C | ctl | OK | 2,31884 | 0,385292 | -2,58938 | -3,79447 | 0,00005 | 0,00421538 | yes |
| ENSDARG00000043196 | rnasel2 | 22:6263716-6264903 | low1C | ctl | OK | 6,0136 | 0,959942 | -2,64721 | -3,9494 | 0,00005 | 0,00421538 | yes |
| ENSDARG00000019492 | shbg | 12:22389795-22403169 | low1C | ctl | OK | 3,11543 | 0,464251 | -2,74646 | -4,0449 | 0,00005 | 0,00421538 | yes |

| | | | | | | | | | | | | |
|---|---|---|---|---|---|---|---|---|---|---|---|---|
| ENSDARG00000018351 | hpda | 5:2544819-2582076 | low1C | ctl | OK | 16,4833 | 2,3534 | -2,80819 | -6,92359 | 0,00005 | 0,00421538 | yes |
| ENSDARG00000071216 | ENDOD1 (5 of 11) | 19:3359896-3363097 | low1C | ctl | OK | 1,02493 | 0,143916 | -2,83223 | -2,98292 | 0,0006 | 0,0340505 | yes |
| ENSDARG00000071076 | ldhbb | 25:3201140-3210843 | low1C | ctl | OK | 11,5261 | 1,60021 | -2,84857 | -4,07473 | 0,00005 | 0,00421538 | yes |
| ENSDARG00000092858 | si:ch1073-126c3.2 | 13:20404176-20409808 | low1C | ctl | OK | 2,42633 | 0,327437 | -2,88949 | -2,7914 | 0,0001 | 0,00772511 | yes |
| ENSDARG00000038439 | fabp10a | 16:52626256-52631239 | low1C | ctl | OK | 452,537 | 53,2093 | -3,08829 | -6,06516 | 0,00005 | 0,00421538 | yes |
| ENSDARG00000019294 | cbln8 | 7:73231372-73233805 | low1C | ctl | OK | 22,2043 | 2,3374 | -3,24786 | -6,33203 | 0,00005 | 0,00421538 | yes |
| ENSDARG00000098898 | si:dkey-20i20.15 | 22:1474765-1476208 | low1C | ctl | OK | 192,988 | 0,786666 | -7,93855 | -4,31097 | 0,00005 | 0,00421538 | yes |

**Table S5:** Complete list of significantly altered Gene Ontologies for biological processes, molecular function and cellular components for differentially expressed genes in low 1-C compared to control $F_1$ embryos. The significant GO terms with altered genes, p-value and Benjamini.

| **Biological process** | | | **Genes** | | **P-value** | **Benjamini** |
|---|---|---|---|---|---|---|
| **Inflammatory and immune response** | | | 29 | | | |
| 1 | GOTERM_BP_FAT | GO:0009611~response to wounding | 25 | F11R, MASP2, C3, CFB, F13A1, C5, SERPING1, CDO1, PLG, AHSG, C8A, FGG, APOA2, LYVE1, FGA, F5, SERPINF2, FGB, HMOX1, F2, SERPINC1, CFH, SERPINA1, C2, SERPIND1 | 3,05E-012 | 3,97E-009 |
| 2 | GOTERM_BP_FAT | GO:0002526~acute inflammatory response | 13 | C8A, APOA2, CFB, SERPINF2, C3, MASP2, F2, C5, CFH, SERPING1, SERPINA1, C2, AHSG | 1,65E-011 | 1,07E-008 |
| 8 | GOTERM_BP_FAT | GO:0006956~complement activation | 8 | C8A, C3, MASP2, CFB, C5, CFH, SERPING1, C2 | 3,64E-008 | 6,77E-006 |
| 9 | GOTERM_BP_FAT | GO:0002541~activation of plasma proteins involved in acute inflammatory response | 8 | C8A, C3, MASP2, CFB, C5, CFH, SERPING1, C2 | 4,32E-008 | 7,03E-006 |
| 10 | GOTERM_BP_FAT | GO:0006954~inflammatory response | 16 | F11R, MASP2, C3, CFB, C5, SERPING1, CDO1, AHSG, C8A, APOA2, SERPINF2, HMOX1, F2, CFH, SERPINA1, C2 | 4,32E-008 | 6,25E-006 |
| 11 | GOTERM_BP_FAT | GO:0006957~complement activation, alternative pathway | 6 | C8A, C3, CFB, C5, CFH, C2 | 8,33E-008 | 1,08E-005 |
| 14 | GOTERM_BP_FAT | GO:0042060~wound healing | 12 | FGG, FGA, F5, FGB, HMOX1, F13A1, F2, SERPINC1, SERPING1, SERPINA1, SERPIND1, PLG | 3,49E-007 | 3,79E-005 |
| 18 | GOTERM_BP_FAT | GO:0006952~defense response | 19 | TCIRG1, F11R, CFB, MASP2, C3, C5, SERPING1, CDO1, AHSG, APOA4, C8A, APOA2, SERPINF2, HMOX1, F2, CFH, SERPINA1, C2, APOM | 1,62E-006 | 1,41E-004 |
| 21 | GOTERM_BP_FAT | GO:0006958~complement activation, classical pathway | 6 | C8A, C3, MASP2, C5, SERPING1, C2 | 3,01E-006 | 2,30E-004 |
| 22 | GOTERM_BP_FAT | GO:0006959~humoral immune response | 8 | C8A, C3, MASP2, CFB, C5, CFH, SERPING1, C2 | 3,07E-006 | 2,22E-004 |
| 23 | GOTERM_BP_FAT | GO:0002455~humoral immune response mediated by circulating immunoglobulin | 6 | C8A, C3, MASP2, C5, SERPING1, C2 | 4,26E-006 | 2,91E-004 |
| 26 | GOTERM_BP_FAT | GO:0002253~activation of immune response | 8 | C8A, C3, MASP2, CFB, C5, CFH, SERPING1, C2 | 9,83E-006 | 5,81E-004 |
| 30 | GOTERM_BP_FAT | GO:0045087~innate immune response | 9 | C8A, APOA4, C3, MASP2, CFB, C5, CFH, SERPING1, C2 | 1,43E-005 | 7,43E-004 |
| 35 | GOTERM_BP_FAT | GO:0050778~positive regulation of immune response | 9 | C8A, C3, MASP2, CFB, HPX, C5, CFH, SERPING1, C2 | 2,05E-005 | 9,51E-004 |
| 48 | GOTERM_BP_FAT | GO:0016064~immunoglobulin mediated immune response | 6 | C8A, C3, MASP2, C5, SERPING1, C2 | 6,85E-005 | 2,22E-003 |
| 50 | GOTERM_BP_FAT | GO:0019724~B cell mediated immunity | 6 | C8A, C3, MASP2, C5, SERPING1, C2 | 8,16E-005 | 2,53E-003 |
| 51 | GOTERM_BP_FAT | GO:0002252~immune effector process | 8 | C8A, C3, MASP2, CFB, C5, CFH, SERPING1, C2 | 9,71E-005 | 2,93E-003 |

| | | | | | | |
|---|---|---|---|---|---|---|
| 57 | GOTERM_BP_FAT | GO:0002449~lymphocyte mediated immunity | 6 | C8A, C3, MASP2, C5, SERPING1, C2 | 2,37E-004 | 6,27E-003 |
| 64 | GOTERM_BP_FAT | GO:0002460~adaptive immune response based on somatic recombination of immune receptors built from immunoglobulin superfamily domains | 6 | C8A, C3, MASP2, C5, SERPING1, C2 | 3,70E-004 | 8,56E-003 |
| 65 | GOTERM_BP_FAT | GO:0002250~adaptive immune response | 6 | C8A, C3, MASP2, C5, SERPING1, C2 | 3,70E-004 | 8,56E-003 |
| **Oxidation reduction** | | | 29 | | | |
| 3 | GOTERM_BP_FAT | GO:0055114~oxidation reduction | 23 | ALDH8A1, GPD1, LDHB, TYRP1, HSD3B7, SCD, FADS2, DHRS9, HGD, NDUFA13, PAH, GRHPR, CDO1, SOD3, F5, HMOX1, TXN, MIOX, HSD11B2, HSD17B4, CAT, QSOX1, SARDH | 5,04E-009 | 2,19E-006 |
| 63 | GOTERM_BP_FAT | GO:0006979~response to oxidative stress | 8 | APOA4, EPAS1, APOE, UCP2, HMOX1, SEPP1, CAT, TAT | 3,38E-004 | 7,97E-003 |
| **Blood coagulation, hemostasis, wound healing** | | | 12 | | | |
| 4 | GOTERM_BP_FAT | GO:0050817~coagulation | 11 | FGG, FGA, F5, FGB, F13A1, F2, SERPINC1, SERPING1, SERPINA1, SERPIND1, PLG | 7,96E-009 | 2,59E-006 |
| 5 | GOTERM_BP_FAT | GO:0007596~blood coagulation | 11 | FGG, FGA, F5, FGB, F13A1, F2, SERPINC1, SERPING1, SERPINA1, SERPIND1, PLG | 7,96E-009 | 2,59E-006 |
| 6 | GOTERM_BP_FAT | GO:0007599~hemostasis | 11 | FGG, FGA, F5, FGB, F13A1, F2, SERPINC1, SERPING1, SERPINA1, SERPIND1, PLG | 1,39E-008 | 3,62E-006 |
| 7 | GOTERM_BP_FAT | GO:0050878~regulation of body fluid levels | 12 | FGG, FGA, F5, FGB, F13A1, F2, SERPINC1, HSD11B2, SERPING1, SERPINA1, SERPIND1, PLG | 1,55E-008 | 3,36E-006 |
| **Processing of proteins, carboxylic- and amino acids** | | | 20 | | | |
| 12 | GOTERM_BP_FAT | GO:0046395~carboxylic acid catabolic process | 10 | PLA2G15, HAL, HGD, PAH, HSD17B4, UROC1, CDO1, TAT, SARDH, CPT1A | 2,41E-007 | 2,86E-005 |
| 13 | GOTERM_BP_FAT | GO:0016054~organic acid catabolic process | 10 | PLA2G15, HAL, HGD, PAH, HSD17B4, UROC1, CDO1, TAT, SARDH, CPT1A | 2,41E-007 | 2,86E-005 |
| 24 | GOTERM_BP_FAT | GO:0051605~protein maturation by peptide bond cleavage | 8 | C8A, C3, MASP2, CFB, C5, CFH, SERPING1, C2 | 5,44E-006 | 3,54E-004 |
| 31 | GOTERM_BP_FAT | GO:0009063~cellular amino acid catabolic process | 7 | HAL, HGD, PAH, UROC1, CDO1, TAT, SARDH | 1,65E-005 | 8,25E-004 |
| 37 | GOTERM_BP_FAT | GO:0016485~protein processing | 8 | C8A, C3, MASP2, CFB, C5, CFH, SERPING1, C2 | 3,09E-005 | 0,00134 |
| 39 | GOTERM_BP_FAT | GO:0009310~amine catabolic process | 7 | HAL, HGD, PAH, UROC1, CDO1, TAT, SARDH | 3,63E-005 | 1,47E-003 |
| 46 | GOTERM_BP_FAT | GO:0051604~protein maturation | 8 | C8A, C3, MASP2, CFB, C5, CFH, SERPING1, C2 | 5,36E-005 | 0,00183 |
| **Lipid transport and metabolic processing (eg. cholesterol)** | | | 28 | | | |
| 15 | GOTERM_BP_FAT | GO:0030301~cholesterol transport | 7 | APOA4, APOA2, APOB, NPC2, APOE, ABCG4, APOM | 4,28E-007 | 4,28E-005 |
| 16 | GOTERM_BP_FAT | GO:0015918~sterol transport | 7 | APOA4, APOA2, APOB, NPC2, APOE, ABCG4, APOM | 4,28E-007 | 4,28E-005 |

| | | | | | | |
|---|---|---|---|---|---|---|
| 17 | GOTERM_BP_FAT | GO:0010035~response to inorganic substance | 12 | APOA4, FGG, APOB, FGA, APOE, FGB, UCP2, HMOX1, SERPINA1, CAT, TAT, SOD3 | 7,07E-007 | 6,57E-005 |
| 19 | GOTERM_BP_FAT | GO:0065005~protein-lipid complex assembly | 5 | APOA4, APOA2, APOB, APOE, APOM | 1,81E-006 | 1,47E-004 |
| 20 | GOTERM_BP_FAT | GO:0034377~plasma lipoprotein particle assembly | 5 | APOA4, APOA2, APOB, APOE, APOM | 1,81E-006 | 1,47E-004 |
| 25 | GOTERM_BP_FAT | GO:0034381~lipoprotein particle clearance | 5 | APOA2, APOB, APOE, HMOX1, APOM | 6,49E-006 | 4,02E-004 |
| 27 | GOTERM_BP_FAT | GO:0042632~cholesterol homeostasis | 6 | APOA4, APOA2, APOB, NPC2, APOE, APOM | 1,20E-005 | 6,80E-004 |
| 28 | GOTERM_BP_FAT | GO:0055092~sterol homeostasis | 6 | APOA4, APOA2, APOB, NPC2, APOE, APOM | 1,20E-005 | 6,80E-004 |
| 29 | GOTERM_BP_FAT | GO:0033344~cholesterol efflux | 5 | APOA4, APOA2, APOE, ABCG4, APOM | 1,36E-005 | 7,36E-004 |
| 32 | GOTERM_BP_FAT | GO:0034369~plasma lipoprotein particle remodeling | 5 | APOA4, APOA2, APOB, APOE, APOM | 1,69E-005 | 8,12E-004 |
| 33 | GOTERM_BP_FAT | GO:0034367~macromolecular complex remodeling | 5 | APOA4, APOA2, APOB, APOE, APOM | 1,69E-005 | 8,12E-004 |
| 34 | GOTERM_BP_FAT | GO:0034368~protein-lipid complex remodeling | 5 | APOA4, APOA2, APOB, APOE, APOM | 1,69E-005 | 8,12E-004 |
| 36 | GOTERM_BP_FAT | GO:0006641~triglyceride metabolic process | 6 | APOA4, APOA2, APOB, APOE, CAT, LIPF | 2,23E-005 | 1,00E-003 |
| 38 | GOTERM_BP_FAT | GO:0008202~steroid metabolic process | 10 | GC, APOA4, APOA2, APOB, HSD3B7, APOE, HSD11B2, DHRS9, CAT, SCP2 | 3,37E-005 | 1,41E-003 |
| 41 | GOTERM_BP_FAT | GO:0006639~acylglycerol metabolic process | 6 | APOA4, APOA2, APOB, APOE, CAT, LIPF | 4,26E-005 | 1,63E-003 |
| 42 | GOTERM_BP_FAT | GO:0046486~glycerolipid metabolic process | 9 | APOA4, GPD1, APOA2, APOB, PLA2G15, APOE, PCYT1B, CAT, LIPF | 4,54E-005 | 1,69E-003 |
| 43 | GOTERM_BP_FAT | GO:0006638~neutral lipid metabolic process | 6 | APOA4, APOA2, APOB, APOE, CAT, LIPF | 4,70E-005 | 1,70E-003 |
| 44 | GOTERM_BP_FAT | GO:0006662~glycerol ether metabolic process | 6 | APOA4, APOA2, APOB, APOE, CAT, LIPF | 5,18E-005 | 1,82E-003 |
| 45 | GOTERM_BP_FAT | GO:0055088~lipid homeostasis | 6 | APOA4, APOA2, APOB, NPC2, APOE, APOM | 5,18E-005 | 1,82E-003 |
| 47 | GOTERM_BP_FAT | GO:0018904~organic ether metabolic process | 6 | APOA4, APOA2, APOB, APOE, CAT, LIPF | 6,25E-005 | 2,08E-003 |
| 49 | GOTERM_BP_FAT | GO:0016042~lipid catabolic process | 9 | APOA4, APOA2, APOB, PLA2G15, APOE, PLA2G12B, HSD17B4, CPT1A, LIPF | 7,22E-005 | 2,29E-003 |
| 52 | GOTERM_BP_FAT | GO:0034375~high-density lipoprotein particle remodeling | 4 | APOA4, APOA2, APOE, APOM | 1,03E-004 | 3,05E-003 |
| 53 | GOTERM_BP_FAT | GO:0006869~lipid transport | 8 | APOA4, APOA2, APOB, NPC2, APOE, ABCG4, APOM, SCP2 | 1,59E-004 | 4,59E-003 |
| 54 | GOTERM_BP_FAT | GO:0045940~positive regulation of steroid metabolic process | 4 | APOA4, APOA2, APOE, ABCG4 | 1,69E-004 | 4,77E-003 |
| 55 | GOTERM_BP_FAT | GO:0019218~regulation of steroid metabolic process | 5 | APOA4, APOA2, APOB, APOE, ABCG4 | 1,86E-004 | 5,13E-003 |
| 58 | GOTERM_BP_FAT | GO:0043691~reverse cholesterol transport | 4 | APOA4, APOA2, APOE, APOM | 2,57E-004 | 6,66E-003 |
| 59 | GOTERM_BP_FAT | GO:0010876~lipid localization | 8 | APOA4, APOA2, APOB, NPC2, APOE, ABCG4, APOM, SCP2 | 2,59E-004 | 6,60E-003 |

| | | | | | | |
|---|---|---|---|---|---|---|
| 60 | GOTERM_BP_FAT | GO:0019216~regulation of lipid metabolic process | 7 | APOA4, APOA2, APOB, NPC2, APOE, ABCG4, APOM | 2,71E-004 | 6,77E-003 |
| **Other significant processes** | | | | | | |
| 40 | GOTERM_BP_FAT | GO:0019748~secondary metabolic process | 7 | ALDH8A1, GPD1, LDHB, TYRP1, DHRS9, RPIA, OCA2 | 3,90E-005 | 1,54E-003 |
| 56 | GOTERM_BP_FAT | GO:0016052~carbohydrate catabolic process | 7 | GPD1, LDHB, LYVE1, GNPDA1, PKLR, MIOX, RPIA | 2,34E-004 | 0,00633 |
| 61 | GOTERM_BP_FAT | GO:0032101~regulation of response to external stimulus | 8 | C3, APOE, SERPINF2, F2, SERPINC1, SERPING1, PLG, AHSG | 2,80E-004 | 0,00686 |
| 62 | GOTERM_BP_FAT | GO:0042592~homeostatic process | 17 | EPAS1, HCLS1, TRIM10, PLG, APOA4, APOA2, APOB, HSP90B1, NPC2, HPX, APOE, HMOX1, F2, TXN, RGN, APOM, QSOX1 | 2,93E-004 | 7,03E-003 |

| **Molecular function** | | | Genes | | P-value | Benjamini |
|---|---|---|---|---|---|---|
| **Peptidase inhibitor activity** | | | 14 | | | |
| 1 | GOTERM_MF_FAT | GO:0004866~endopeptidase inhibitor activity | 13 | C3, FETUB, C5, SERPING1, AHSG, AMBP, RENBP, SERPINF2, SERPINC1, SERPINA1, ITIH2, SERPIND1, ITIH3 | 1,91E-009 | 6,21E-007 |
| 2 | GOTERM_MF_FAT | GO:0030414~peptidase inhibitor activity | 13 | C3, FETUB, C5, SERPING1, AHSG, AMBP, RENBP, SERPINF2, SERPINC1, SERPINA1, ITIH2, SERPIND1, ITIH3 | 3,54E-009 | 5,75E-007 |
| 3 | GOTERM_MF_FAT | GO:0004857~enzyme inhibitor activity | 14 | C3, FETUB, C5, SERPING1, AHSG, AMBP, APOA2, RENBP, SERPINF2, SERPINC1, SERPINA1, ITIH2, SERPIND1, ITIH3 | 2,59E-007 | 2,80E-005 |
| 6 | GOTERM_MF_FAT | GO:0004867~serine-type endopeptidase inhibitor activity | 8 | AMBP, SERPINF2, SERPINC1, SERPING1, ITIH2, SERPINA1, SERPIND1, ITIH3 | 9,22E-006 | 4,99E-004 |
| **Lipid transport (especially cholesterol)** | | | 15 | | | |
| 4 | GOTERM_MF_FAT | GO:0015248~sterol transporter activity | 5 | APOA4, APOA2, APOB, APOE, HSD17B4 | 3,80E-006 | 3,09E-004 |
| 11 | GOTERM_MF_FAT | GO:0017127~cholesterol transporter activity | 4 | APOA4, APOA2, APOB, APOE | 1,07E-004 | 3,48E-003 |
| 12 | GOTERM_MF_FAT | GO:0005319~lipid transporter activity | 6 | APOA4, APOA2, APOB, APOE, HSD17B4, APOM | 1,52E-004 | 4,49E-003 |
| 13 | GOTERM_MF_FAT | GO:0008201~heparin binding | 7 | APOB, APOE, SERPINC1, VTN, SERPIND1, COL5A3, SOD3 | 1,84E-004 | 4,96E-003 |
| 14 | GOTERM_MF_FAT | GO:0070325~lipoprotein receptor binding | 4 | APOA2, APOB, HSP90B1, APOE | 2,18E-004 | 5,43E-003 |
| 15 | GOTERM_MF_FAT | GO:0043499~eukaryotic cell surface binding | 4 | APOA4, FGG, FGA, FGB | 3,22E-004 | 7,45E-003 |
| **Protein remodelling** | | | 17 | | | |
| 5 | GOTERM_MF_FAT | GO:0046983~protein dimerization activity | 17 | SHBG, GPD1, TYRP1, CNDP1, EPAS1, CNDP2, PAH, MYH6, MYH9, ABCG4, AMBP, APOA4, APOA2, APOB, RENBP, APOE, CAT | 6,82E-006 | 4,43E-004 |
| 7 | GOTERM_MF_FAT | GO:0042803~protein homodimerization activity | 13 | SHBG, GPD1, TYRP1, PAH, MYH6, MYH9, ABCG4, APOA4, AMBP, APOA2, RENBP, APOE, CAT | 1,58E-005 | 7,31E-004 |

| | | | | | | |
|---|---|---|---|---|---|---|
| **Other significant processes** | | | 9 | | | |
| 8 | GOTERM_MF_FAT | GO:0005539~glycosaminoglycan binding | 9 | LYVE1, APOB, APOE, SERPINC1, VTN, SERPIND1, COL5A3, SOD3, HABP2 | 1,73E-005 | 7,03E-004 |
| 9 | GOTERM_MF_FAT | GO:0030247~polysaccharide binding | 9 | LYVE1, APOB, APOE, SERPINC1, VTN, SERPIND1, COL5A3, SOD3, HABP2 | 3,44E-005 | 0,00124 |
| 10 | GOTERM_MF_FAT | GO:0001871~pattern binding | 9 | LYVE1, APOB, APOE, SERPINC1, VTN, SERPIND1, COL5A3, SOD3, HABP2 | 3,44E-005 | 0,00124 |

| **Cellular component** | | | **Genes** | | **P-value** | **Benjamini** |
|---|---|---|---|---|---|---|
| **Extracellular space and region** | | | 49 | | | |
| 1 | GOTERM_CC_FAT | GO:0005576~extracellular region | 49 | CNDP1, MASP2, F13A1, VTN, APOA4, APOA2, APOB, APOE, HMOX1, CFH, ITIH2, SERPINA1, ITIH3, SEPP1, APOM, SHBG, SERPING1, CTSS, CCDC134, AMBP, C8A, NPC2, F5, SERPINF2, F2, VMO1, GC, C3, C5, CPN2, AHSG, FGG, FGA, FGB, PLA2G12B, SERPINC1, C2, QSOX1, PLA2G15, CFB, FETUB, ENDOD1, COL5A3, PLG, SOD3, HPX, SERPIND1, HABP2, LIPF | 1,32E-013 | 2,70E-011 |
| 2 | GOTERM_CC_FAT | GO:0005615~extracellular space | 29 | GC, C3, C5, VTN, AHSG, APOA4, APOB, APOA2, FGG, FGA, APOE, FGB, HMOX1, SERPINC1, CFH, SERPINA1, SEPP1, C2, APOM, QSOX1, SERPING1, PLG, SOD3, C8A, F5, HPX, SERPINF2, F2, HABP2 | 4,36E-013 | 4,47E-011 |
| 3 | GOTERM_CC_FAT | GO:0044421~extracellular region part | 30 | GC, C3, C5, VTN, AHSG, APOA4, APOB, APOA2, FGG, FGA, APOE, FGB, HMOX1, SERPINC1, CFH, SERPINA1, SEPP1, C2, APOM, QSOX1, SERPING1, COL5A3, PLG, SOD3, C8A, F5, HPX, SERPINF2, F2, HABP2 | 2,53E-010 | 1,73E-008 |
| **Vesicle lumen, platelet - and secretory granules** | | | 9 | | | |
| 4 | GOTERM_CC_FAT | GO:0031983~vesicle lumen | 9 | FGG, APOB, FGA, F5, FGB, SERPINF2, F13A1, SERPING1, SERPINA1 | 2,73E-009 | 1,40E-007 |
| 5 | GOTERM_CC_FAT | GO:0031093~platelet alpha granule lumen | 8 | FGG, FGA, F5, FGB, SERPINF2, F13A1, SERPING1, SERPINA1 | 3,22E-008 | 1,32E-006 |
| 6 | GOTERM_CC_FAT | GO:0060205~cytoplasmic membrane-bounded vesicle lumen | 8 | FGG, FGA, F5, FGB, SERPINF2, F13A1, SERPING1, SERPINA1 | 5,39E-008 | 1,84E-006 |
| 7 | GOTERM_CC_FAT | GO:0031091~platelet alpha granule | 8 | FGG, FGA, F5, FGB, SERPINF2, F13A1, SERPING1, SERPINA1 | 3,01E-007 | 8,82E-006 |
| 15 | GOTERM_CC_FAT | GO:0030141~secretory granule | 8 | FGG, FGA, F5, FGB, SERPINF2, F13A1, SERPING1, SERPINA1 | 6,21E-004 | 0,00975 |
| **Lipid -protein complex (e.g. lipoprotein)** | | | 5 | | | |
| 8 | GOTERM_CC_FAT | GO:0034385~triglyceride-rich lipoprotein particle | 5 | APOA4, APOA2, APOB, APOE, APOM | 1,74E-005 | 4,47E-004 |

| | | | | | | |
|---|---|---|---|---|---|---|
| 9 | GOTERM_CC_FAT | GO:0034361~very-low-density lipoprotein particle | 5 | APOA4, APOA2, APOB, APOE, APOM | 1,74E-005 | 4,47E-004 |
| 11 | GOTERM_CC_FAT | GO:0042627~chylomicron | 4 | APOA4, APOA2, APOB, APOE | 1,06E-004 | 2,17E-003 |
| 12 | GOTERM_CC_FAT | GO:0034358~plasma lipoprotein particle | 5 | APOA4, APOA2, APOB, APOE, APOM | 1,72E-004 | 3,20E-003 |
| 13 | GOTERM_CC_FAT | GO:0032994~protein-lipid complex | 5 | APOA4, APOA2, APOB, APOE, APOM | 1,72E-004 | 3,20E-003 |
| **Lysosomes and vacuoles** | | | 10 | | | |
| 10 | GOTERM_CC_FAT | GO:0044433~cytoplasmic vesicle part | 10 | FGG, TYRP1, APOB, FGA, F5, FGB, SERPINF2, F13A1, SERPING1, SERPINA1 | 1,95E-005 | 4,44E-004 |
| **Endoplasmatic reticulum and mitochondrion** | | | 6 | | | |
| 14 | GOTERM_CC_FAT | GO:0005788~endoplasmic reticulum lumen | 6 | APOA4, CES3, APOA2, APOB, HSP90B1, CALR3 | 4,59E-004 | 7,81E-003 |

**Table S6:** Enrichment of KEGG pathway members in the differentially expressed genes in low 1-C embryos compared to control embryos. The five most enriched pathways are listed.

| | **Kegg pathways** | **Genes** | | **P-value** | **Benjamini** |
|---|---|---|---|---|---|
| 1 | hsa04610:Complement and coagulation cascades | 19 | CFB, MASP2, C3, F13A1, C5, SERPING1, PLG, C8A, FGG, F5, FGA, FGB, SERPINF2, F2, SERPINC1, CFH, SERPINA1, SERPIND1, C2 | 6,92E-019 | 5,81E-017 |
| 2 | hsa03320:PPAR signalling pathway | 6 | APOA2, SCD, FADS2, UCP1, SCP2, CPT1A | 2,92E-003 | 1,16E-001 |
| 3 | hsa00340:Histidine metabolism | 4 | CNDP1, FTCD, HAL, UROC1 | 7,94E-003 | 2,00E-001 |
| 4 | hsa00270:Cysteine and methionine metabolism | 4 | LDHB, MAT1A, CDO1, TAT | 1,23E-002 | 2,30E-001 |
| 5 | hsa00120:Primary bile acid biosynthesis | 3 | HSD3B7, HSD17B4, SCP2 | 1,71E-001 | 3,05E-001 |
